# The epidemiological characteristic and trends of burns globally

**DOI:** 10.1186/s12889-022-13887-2

**Published:** 2022-08-22

**Authors:** Aobuliaximu Yakupu, Jie Zhang, Wei Dong, Fei Song, Jiaoyun Dong, Shuliang Lu

**Affiliations:** 1 Wound Healing Center, Ruijin Hospital, Shanghai Jiao Tong University School of Medicine, Shanghai, China; 2 Department of Burn, Ruijin Hospital, Shanghai Jiao Tong University School of Medicine, Shanghai, China; 3 Shanghai Burn Institute, Ruijin Hospital, Shanghai JiaoTong University School of Medicine, No. 197 Ruijin Er Road, Shanghai, 200025 China

**Keywords:** Burns, Burden, Trends, Epidemiology

## Abstract

**Background:**

Burns is a type of injury, caused by unintentional exposure to substances of high temperature, including hot liquid, solid, and objects radiating heat energy, placing a high burden not only on patients’ families but also on national healthcare systems globally. It is difficult for policymakers and clinicians to formulate targeted management strategies for burns because data on current epidemiological patterns worldwide are lacking.

**Methods:**

Data on burns were obtained from the Global Burden of Disease (GBD) 2019 Study. The incidence, disability-adjusted life years (DALYs), and deaths of burns in 204 countries and regions from 1990 to 2019 were calculated and stratified by sex, age, geographical location, and sociodemographic index (SDI). The estimated annual percentage change (EAPC) of incidence, DALYs, and deaths was calculated to evaluate the temporal trends. All analyses were performed using R software, version 4.1.1, with 2-sided *P*-values < .05 indicating a statistically significant difference.

**Results:**

A total of 8,378,122 new cases (95% UI, 6,531,887–10,363,109cases) of burns were identified globally in 2019, which is almost evenly split between men and women, and most of the new cases were concentrated in the 10–19-year age group. Besides, burns account for 111,292 deaths (95% UI, 132,392–88,188) globally in 2019, most of which were concentrated in those aged 1–4 years. The burden of burns measured in DALYs was 7,460,448.65 (95% UI, 5,794,505.89–9,478,717.81) in 2019, of which 67% and 33% could be attributed to YLLs and YLDs, respectively. The EAPC of incidence, DALYs, and deaths were negative, the age-standardized rate (ASR) of incidence, DALYs, and deaths were considered to be decreasing in most of the regions, and the EAPCs were negatively correlated with SDI levels, universal health coverage (UHC), and gross domestic product (GDP).

**Conclusion:**

Globally, the age-standardized rates of burn incidence, DALYs, and mortality, as well as the number of burn DALYs and death cases will continuously decrease, but the number of new burn cases has an increasing tendency globally. In addition, the EAPCs of burns in incidence, DALYs, and deaths indicated that the burden of burns was considered to be decreasing in most of the regions. And from the relationship of EAPCs with SDI, UHC index, and GDP, indicate that prevention burns not only depend on health spending per capita but also depend on the education level per capita and healthcare system performance, but it does not mean higher health spending corresponds to higher UHC index, which needs high efficiency of translating health spending into individuals health gains.

**Supplementary Information:**

The online version contains supplementary material available at 10.1186/s12889-022-13887-2.

## Background

Burns is a type of injury, caused by unintentional exposure to substances of high temperature, including hot liquid, solid, or gas such as cooking stoves, smoke, steam, drinks, machinery, appliances, tools, radiators, and objects radiating heat energy [1]. Burn injuries can lead to long-term profound alterations even the wounds have healed, which affects not only the physical health but also the mental health and quality of life of the patient, placing a high burden not only on patients’ families but also on national healthcare systems globally [2–4].

The knowledge about burn epidemiology is essential for resource allocation and prevention, but the previous works mainly focused on a specific country or region, which led to a lack of epidemiological information on burns at the global level, and the results are not comparable because of the variable and inconsistent dates have not been standardized; In addition, there is no multi-level and multi-angle analysis on burns epidemiological characters [4–9]. Therefore, it is necessary to understand the latest spatial distribution and temporal trends of burns worldwide to establish more reasonable and effective prevention and treatment programs to improve patients’ quality of life and reduce avoidable medical expenses.

To investigate the level, trends, and burden of burns at the national, regional, and global levels, to identify the risk factors and potential influences of economic income and health services coverage on disease prevalence, we have extracted and analyzed annual data on burns incidence, disability-adjusted life years (DALYs) and deaths by location, sex, and age, as well as risk factors from behavioral, environmental and occupational, and metabolic aspects [10]. This study will provide a basis for optimizing strategies for the management of burns.

## Materials and methods

We collected annual case data and age-standardized rates (ASRs) for burns incidence, deaths, and DALYs from 1990 to 2019 from the Institute for Health Metrics and Evaluation using the Global Health Data Exchange (GHDx) online query tool (http://ghdx.healthdata.org/gbd-results-tool) [1].

The detailed original data introduction and general analysis methods of the GBD 2019 Study have been described in previous research [1, 10–13]. Briefly, the GBD estimation process is based on identifying multiple relevant data sources for each disease or injury and correcting the known bias. Then, the processed data are modeled using standardized tools to generate estimates of each quantity of interest by age, sex, location, and year. There are three main standardized tools: Cause of Death Ensemble model (CODEm) which is a highly systematized tool to analyze the cause of death data, spatiotemporal Gaussian process regression (ST-GPR) that borrow strength between locations and over time for single metrics of interest, and DisMod-MR that is a Bayesian meta-regression tool that allows evaluation of all available data on incidence, prevalence, remission, and mortality for a disease. ASRs were calculated by adjusting for population size (per 100 000 population) and for age structure. We further calculated the estimated annual percentage change (EAPC) to describe the temporal trend in various age-standardized rates (ASRs) of burns burden and the detailed methods have been described in previous research [14–17]. we analyzed a set of behavioral, environmental and occupational, and metabolic risks that contribute to health outcomes. Which was evaluated in GBD 2019 [10].

The 95% uncertainty intervals (UIs) for every metric in the 2019 GBD study were calculated to reflect the certainty of the estimates, which were determined by the 25^th^ and 975^th^ values of the 1,000 values, after ordering them from smallest to largest [1, 10, 18]. We also calculated 95% confidence intervals (95%CIs) for the EAPCs [19]. Temporal trends in ASRs were recognized to be in an increasing trend when the EAPCs and the lower boundary of the 95% CI were positive; conversely, to be a decreasing trend when EAPCs and the upper boundary of the 95% CI were negative. Otherwise, the ASRs were considered to be stable [19, 20]. The data that we extracted from GBD 2019 were collected from 204 countries and territories (data sources including censuses, household surveys, civil registration and vital statistics, disease registries, health service use, air pollution monitors, satellite imaging, disease notifications, and other sources) and were divided into five regions according to their sociodemographic index (SDI) that was developed by GBD researchers and is a composite indicator constructed from measures of per capita income, average years of education, and total fertility rates. In short, SDI is the geometric mean of 0 to 1 index of total fertility rate (TFR) for those younger than 25 years old (TFU25), mean education for those 15 years old and older (EDU15 +), and lag distributed income (LDI) per capita. For GBD 2019, after calculating SDI, values were multiplied by 100 for a scale of 0 to 100. Geographically, the 204 countries and territories were further classified into 45 regions by their location and the detailed information can be seen in the supplementary material (Supplemental Table 1). Besides, we also analyzed the risk factors relative to burns by MR-BRT and ST-GPR modeling [10].

**Table 1:** Incident Cases, Age-Standardized Incidence Rate (ASIR), and Temporal Trends for Burns From 1990 to 2019

	**No. (95% UI)**				**No. (95% CI)**
	1990		2019		1990–2019
**Variable**	Incident cases	ASIR per 100,000	Incident cases	ASIR per 100,000	EAPC
**Global**	8,378,121.71(6,531,886.66 to 10,363,108.53)	149.86(118.1 to 183.52)	8,955,227.69(6,820,977.02 to 11,157,666.34)	117.51(88.79 to 146.66)	-0.93(-0.82 to -1.03)
**male**	4,444,900.33(3,503,352.17 to 5,467,109.97)	157.69(125.36 to 191.85)	4,520,220.92(3,458,623.54 to 5,616,794.5)	117.04(89.04 to 145.43)	-1.13(-1.04 to -1.22)
**female**	3,933,221.38(3,001,685.66 to 4,896,272.45)	142.08(109.68 to 174.96)	4,435,006.77(3,356,767.64 to 5,533,943.36)	118.26(88.71 to 148.07)	-0.71(-0.59 to -0.82)
**SDI**					
**High**	1,829,400.67(1,449,098.13 to 2,233,743.92)	231.04(182.62 to 281.57)	1,617,342.41(1,242,112.77 to 1,998,030.5)	182.79(137.78 to 229.19)	-1.09(-0.89 to -1.29)
**High-middle**	2,268,203.43(1,767,421.04 to 2,791,394.84)	193.43(151.47 to 235.99)	1,968,714.13(1,520,777.66 to 2,446,137.06)	150.53(113.48 to 188.45)	-0.97(-0.87 to -1.07)
**Middle**	2,318,376.08(1,758,311.51 to 2,925,009.94)	122.15(94.32 to 151.55)	2,489,842.35(1,867,023.57 to 3,119,116.86)	108.16(80.58 to 137.69)	-0.32(-0.21 to -0.43)
**Low-middle**	1,261,052.24(958,368.21 to 1,588,226.97)	100.76(78.12 to 124.86)	1,578,840.04(1,180,460.57 to 2,007,600.31)	84.85(64.03 to 106.79)	-0.69(-0.58 to -0.79)
**Low**	694,078.23(535,540.88 to 876,228.73)	118.29(92.6 to 145.79)	1,291,495.65(947,627.72 to 1,674,261.6)	101.2(77.25 to 127.39)	-0.66(-0.57 to -0.76)

*Abbreviations*: *ASIR* Age-standardized incidence rate, *EAPC* Estimated annual percentage change, *SDI* Sociodemographic index, *UI* Uncertainty interval, *CI* Confidence interval

For exploring the potential factors of changing trends, we also calculated the association between universal health coverage (UHC), gross domestic product (GDP) with EAPCs in burns burden. Achieving universal health coverage (UHC) involves all individuals receiving the health services they need, of high quality, without experiencing financial difficulty and the UHC effective coverage index provides the understanding of whether health services are aligned with countries' health profiles and are of sufficient quality to produce health gains for populations [21].

### Data analysis

EAPCs were calculated on a regression model. The natural logarithm of the regression line is fitted to ASR with the following formula, y = α + β x + ε, where y = ln (ASR), and x = calendar year; The EAPCs 95% CI were calculated based on 100 × [exp(β) − 1] [17–19, 22]. The correlations of EAPCs with UHC in 2019, GDP in 2019, and SDI value using a Pearson correlation analysis [22]. All analyses were performed using R software, version 4.1.1, with 2-sided *P*-values < 0.05 indicating a statistically significant difference.

## Results

### Incidence of burns

**Fig. 1:**
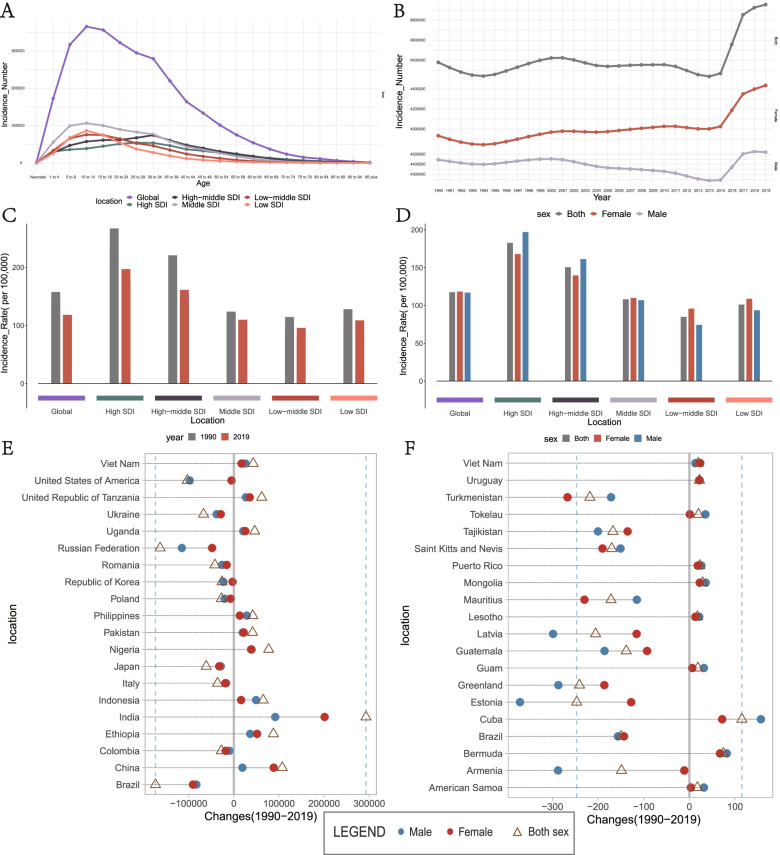
Incidence of burns. **A** distribution of new cases among different age categories in 2019. **B** the global changing trend in the number of new cases by sex from 1990 to 2019. **C** a comparison of the ASIR between 1990 and 2019 at global and different SDI levels. **D** a comparison of the ASIR by sex in 2019 at global and different SDI levels. **E** the top increased or decreased in the number of new cases by sex in 2019 compared with 1990 among 204 countries and territories. **F** the top increased or decreased in ASIR by sex in 2019 compared with 1990 among 204 countries and territories. ASIR, age-standardized incidence rate; SDI, sociodemographic index

Globally, a total of 8,955,228 new cases (95% UI 6,820,977–11157666cases) of burns were identified in 2019, which is almost evenly split between men and women, and most of the new cases were concentrated in the 10–19-year age group (Table 1, Fig. 1A). From 1990 to 2015, the number of incident cases fluctuates within a certain degree, but the number of incident cases sharply increased from 2016 (Table 1, Fig. 1B). Females affected occupied about 87% of the increased cases from 1990 to 2019 (Table 1). However, the age-standardized incidence rate (ASIR) was found to have decreased by an average of 0.7% per year in the same period (from 149.86 per 100,000 in 1990 to 117.51 per 100,000 in 2019) and the ASIR value of both genders is similar in 2019, besides incidence distribution by age can be seen in supplemental materials (Table 1, Supplemental Figure S 1A, D, E).

Among the SDI quintiles, countries with Middle SDI level has the highest incident cases than countries with other SDI levels, both in 1990 and 2019, and the number of incident cases increased in Low, Low-middle, and Middle SDI levels from 1990 to 2019, whereas the number decreased in High and High-middle SDI levels (Table 1, Supplemental Figure S 1B). However, the ASIR decreased for all SDI levels and countries with the High SDI level had the highest ASIR than those with lower SDI levels, both in 1990 and 2019 (Fig. 1C). The number of new cases was higher in females than in males in Low-middle, Low SDI levels than in other SDI levels both in 1990 and 2019 (Fig. 1D, Supplemental Figure S 1B).

The absolute number of burns cases increased in most of GBD regions. Asia had the most new cases in 2019 (3,913,524.80 [95% UI 2,946,199.45–4,959,607.51]), with a 19% increase relative to cases in 1990 (3,287,567.95 [95% UI, 2,473,836.69–4,165,597.49]), whereas Oceania had the fewest new cases in 2019 (27,510.31 [95% UI,21,933.81–33,261.04]). The regions that had the most significantly increased and decreased numbers of new cases, respectively, were World Bank Lower Middle Income category (from 2,118,416.48 [95% UI, 1,626,255.73–2,646,959.73] to 2,827,973.07 [95% UI, 2,124,427.64–3,575,667.26]) and the Europe (from 1,982,378.87 [95% UI, 1,585,062.86–2,411,800.64] to 1,492,624.00 [95% UI,1,169,397.61–1,831,234.57]) Europe (Supplemental Figure S 1I). As for the ASIR, Caribbean had the highest ASIR in 2019 (336.06 [95% UI, 267.46–414.72]), whereas Eastern Mediterranean Region had the lowest (57.43 [95% UI, 44.27–71.52]). The ASIRs decreased in most of the regions. The most greatest reduction detected was in Tropical Latin America (from 286.29 [95% UI, 219.37–352.94] to 140.70 [95% UI, 107.88–175.49]), whereas the most significant increase was detected in East Asia (from 76.47 [95% UI, 57.12–96.95] to 85.93 [95% UI, 61.61–110.91]) (Supplemental Figure S 1J).

**Fig. 2:**
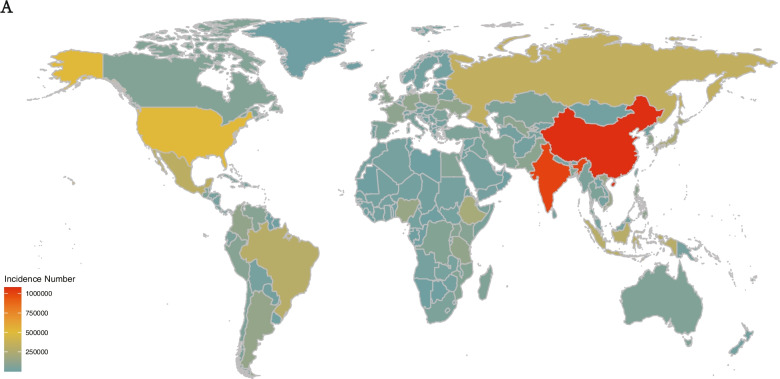
The map of new cases in 2019. **A** the map of new cases in 2019 among 204 countries and territories

At the national level, the most highest number of cases were recorded in Mainland of China in 2019, which account for 12% of the new cases detected globally (overall, 1,079,670.14 [95% UI, 786,947.53–1,389,157.59]; males, 565,448.23 [95% UI, 417,357.69–728,230.85]; females, 514,221.91 [95% UI,370,324.64–660,910.29]) (cases number peaked in those who were 30–34 years of age), followed by India (1,009,518.92 [95% UI, 742,769.05–1,295,202.13]), collectively accounting for 11% of all new cases, globally. Niue had the fewest new cases in 2019 (1.84 [95% UI, 1.35–2.39] cases). The most significant reduction detected was in Brazil (from 463,695.51 [95% UI, 350,241.35–583,046.27] to 290,004.98 [95% UI, 225,525.87–362,276.26]), whereas the most significant increase was detected in India (from 716,858.75 [95% UI, 534,524.91–918,816.44] to 1,009,518.92 [95% UI, 742,769.05–1,295,202.13]), followed by Mainland of China where increased about 972,398 cases (Fig. 1E). Cuba had the highest ASIR in 2019 (overall, 460.33 [95% UI, 347.29–585.47]; males, 483.27 [95% UI, 364.71–615.59); females, 435.87 [95% UI, 326.68–555.02]), and the lowest ASIR was observed in Pakistan(overall, 35.50 [95% UI, 26.20–45.88]; males, 29.48 [95% UI, 21.69–38.07]; females, 41.90 [95% UI, 54.77–30.63]). The regions with the largest increase and decrease in the ASIR were Cuba and Estonia, respectively (Fig. 1F). Furthermore, we visualized the number of cases in 2019 among 204 countries and territories by map (Fig. 2A).

#### DALYs of burns

**Fig. 3:**
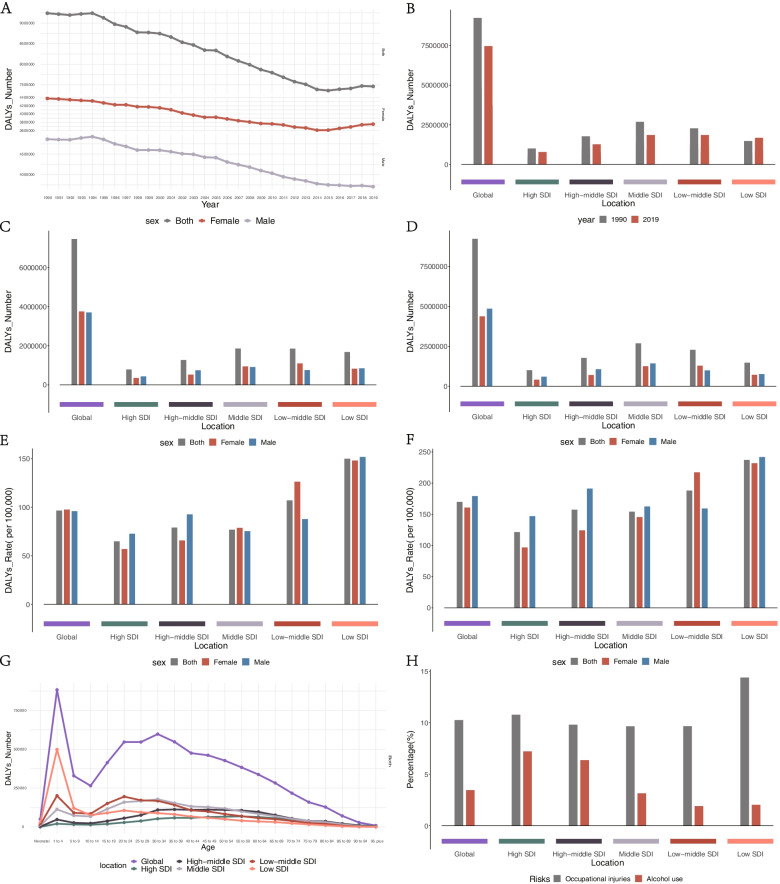
DALYs of burns. **A** the global changing trend in DALYs by sex from 1990 to 2019. **B** a comparison of the DALYs between 1990 and 2019 at global and different SDI levels. **C** a comparison of the DALYs by sex at global and different SDI levels in 2019. **D** a comparison of the DALYs by sex at global and different SDI levels in 1990. **E** a comparison of the ASDAR by sex at global and different SDI levels in 2019. **F** a comparison of the ASDAR by sex at global and different SDI levels in 1990. **G** distribution of DALYs among different age categories in 2019. **H** the percentage of DALYs attributable to top risk factors for both sexes combined at global and different SDI levels in 2019. DALYs, disability-adjusted life years; ASDAR, age-standardized DALYs rate; SDI, sociodemographic index

The burden of burns measured in DALYs was 7,460,448.65 (95% UI, 5,794,505.89–9,478,717.81) in 2019, of which 67% and 33% could be attributed to YLLs and YLDs, respectively. The burden decreased gradually from 1990 to 2019 (Table 2, Fig. 3A). And the age-standardized DALYs rate (ASDAR) also decreased substantially from 1990 to 2019 (Table 2, Supplemental Figure S 2A, B). The DALYs among females was higher than among males, and the ASDAR among females also was higher than among males in 2019, which is just the opposite in 1990 (Table 2, Fig. 3 C, D, E, F). DALYs were high in the younger population and the highest DALYs were observed in the 1–4-year age group both in 2019 and 1990, DALYs rate distribution by age can be seen in supplemental materials (Figure S 2 G). In addition, 15% of DALYs were attributable to risk factors for both sexes combined globally in 2019, of which 10% and 5% could be attributed to occupational risks and alcohol use (Fig. 3 H).

**Table 2:** DALYs, Age-Standardized DALYs Rates and Temporal Trends for Burns From 1990 to 2019

	No. (95% UI)				No. (95% CI)
	1990		2019		1990–2019
Variable	DALYs	ASDAR per 100,000 people	DALYs	ASDAR per 100,000 people	EAPC
Global	9,240,519.41(6,971,954.76 to 11,508,751.2)	169.85(129.91 to 209.27)	7,460,448.65(5,794,505.89 to 9,478,717.81)	96.6(75.03 to 123.05)	-2.13(-2.06 to -2.21)
Male	4,862,036.55(3,630,820.92 to 5,672,841.93)	179.26(138.91 to 209.21)	3,706,456.4(2,900,689.35 to 4,597,302.42)	95.91(75.01 to 119.1)	-2.33(-2.24 to -2.43)
Female	4,378,482.86(2,430,157.83 to 6,087,956.08)	160.84(93.48 to 219.06)	3,753,992.25(2,820,247.72 to 4,897,043.09)	97.63(73.02 to 127.98)	-1.92(-1.84 to -1.99)
SDI					
High	1,008,484.65(822,574.1 to 1,271,676.7)	121.48(101.06 to 150.14)	785,070.61(577,164.47 to 1,104,553.7)	64.9(48.56 to 89.23)	-2.36(-2.17 to -2.56)
High-middle	1,777,696.5(1,525,910.33 to 2,060,491.85)	157.37(135.25 to 182.22)	1,273,402.1(1,031,688.77 to 1,646,553.63)	79.11(64.33 to 100.63)	-2.86(-2.55 to -3.16)
Middle	2,688,458.99(2,001,006.36 to 3,355,364.82)	154.13(116.77 to 189.75)	1,859,086.98(1,385,859.19 to 2,429,347.02)	76.87(57.21 to 100.44)	-2.55(-2.47 to -2.63)
Low-middle	2,280,046.88(1,474,160.34 to 3,095,008.34)	187.95(128.19 to 246.34)	1,855,910.26(1,435,536.98 to 2,276,397.59)	107.04(83.44 to 130.59)	-2.06(-2.01 to -2.12)
Low	1,477,894.5(949,450 to 2,012,908.16)	237.17(168.86 to 306.75)	1,678,780.87(1,268,530.73 to 2,162,104.1)	149.99(114.86 to 188.57)	-1.58(-1.55 to -1.6)

*Abbreviations*: *DALYs* Disability-adjusted life years, *ASDAR* Age-standardized DALYs rate, *EAPC* Estimated annual percentage change, *SDI* Sociodemographic index, *UI* Uncertainty interval, *CI* Confidence interval

Among the SDI quintiles, DALYs were decreased in all SDI levels except the Low SDI level in 2019 compared to those in 1990, and countries with the Middle SDI level had higher DALYs than countries with other SDI levels in 1990 and the Low-middle SDI level in 2019 (Table 2, Fig. 3B). The ASDARs were significantly decreased in regions with all SDI levels, countries with the Low SDI level had higher DALYs than countries with other SDI levels both in 1990 and 2019 (Table 2, Supplemental Figure S2B). Concerning the sex ratios of DALYs and ASDARs among SDI levels, except for the low-middle SDI level, the DALYs and ASDAR were higher in males for all SDI levels in both 1990 and 2019 (Fig. 3C, D, E, F). In 2019, occupational risks are the highest contribution of DALYs in countries with lower SDI levels, while alcohol use is the highest contribution in countries with higher SDI levels (Fig. 3H).

For most of the GBD regions, the absolute DALYs of burns was decreased, with the highest DALYs observed in 2019 is in Asia (3,735,101.26 [95%UI, 2,763,340.35–4,744,685.60]) and the lowest observed in Australasia (20,813.30 [95%UI, 13,842.51–30,776.21); meanwhile, Western Sub-Saharan Africa (from 471,311.76 [95% UI, 311,274.–77,648,837.12] to 670,821.92 [95% UI, 493,686.72–928,527.33]) and World Bank Upper Middle Income regions(from 3,309,575.83 [95% UI, 2,642,128.26–3,932,627.20] to 2,066,166.93 [95% UI, 1,644,020.10–2,697,166.96]) exhibited the most significant increase and decrease in numbers, respectively (Supplemental Figure S2E). As for the ASDAR of burns, for all except one GBD regions, namely Oceania, the rate decreased. The greatest ASDAR was observed in 2019 in the Oceania (472.12 [95%UI, 118.52–780.73])and the lowest was in East Asia (48.28 [95%UI, 34.94–68.00). The most significant decrease in the ASDAR was detected in Central Asia (from 438.56 [95% UI, 393.52–493.45] to 196.25 [95% UI, 163.25–242.53]), whereas the most significant increase was detected in Oceania (from 419.79 [95% UI, 664.47–145.61] to 425.30 [95% UI, 118.75–687.13]) (Supplemental Figure S2F).

For the assessment of changes at the national level, the highest DALYs, accounting for 11% of the value globally, was recorded in India in 2019 (overall, 1,577,243.30 [95% UI, 1,069,291.85–2,223,193.75]; males, (423,295.84 [95% UI, 321,148.61–528,849.46]); females, (28,036.17 [95% UI, 20,767.08–35,787.12])), followed by Mainland of China (1,153,947.45 [95% UI, 711,833.97–1,778,176.96]). In India, the 6% of age-standardized DALYs result from occupational injuries and the DALYs reached a peak among those 20 ~ 24 years of age in 2019. The lowest DALYs were observed in Tokelau (0.92 [95%UI, 0.66–1.31). As for measures of the ASDAR, Papua New Guinea had the highest ASDAR in 2019 (overall, 497.21 [95% UI, 116.10–824.53]); males, (868.96 [95% UI, 180.42–1446.77]); females, (95.77 [95% UI, 40.06–165.90])). Italy had the lowest ASDAR in 2019 (26.83 [95% UI, 19.82–36.37]); males, (32.71 [95% UI, 24.16–44.24]); females, (21.13 [95% UI, 15.50–28.76]). The places that exhibited the most significantly increased and decreased DALY values, respectively, were Nigeria(from 204,791.75 [95% UI, 128,433.17–305,529.32] to 278,416.67 [95% UI, 197,549.76–400,510.66]) and Mainland of China(from 1,204,419.61 [95% UI, 862,545.83–1,493,528.50] to 687,955.14 [95% UI, 494,849.21–973,860.45]) (Supplemental Figure S2G), whereas, for the ASIR, the locations were, respectively, Lesotho (from 272.21 [95% UI, 198.26–375.43] to 336.32 [95% UI, 246.06–442.86]) and Haiti(from 827.26 [95% UI, 324.99–1,258.22] to 385.90 [95% UI, 210.16–525.93]) (Supplemental Figure S2H).

#### Mortality of burns

**Fig. 4:**
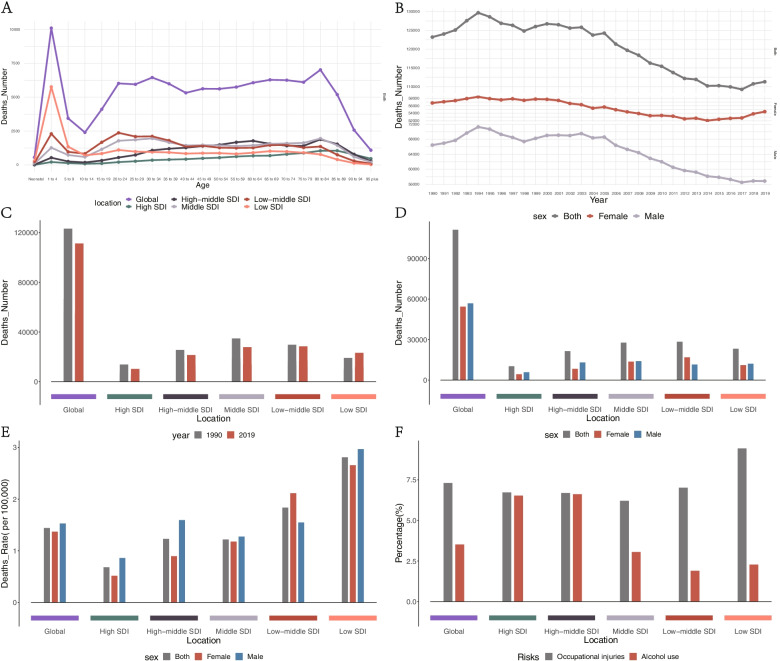
Mortality of burns. **A** distribution of death cases among different age categories in 2019. **B** the global changing trend in the number of death cases by sex from 1990 to 2019. **C** a comparison of the number of death cases between 1990 and 2019 at global and different SDI levels. **D** a comparison of the number of death cases by sex at global and different SDI levels in 2019. **E** a comparison of the ASDR by sex at global and different SDI levels in 2019. **F** Percentage of deaths attributable to top risk factors for both sexes combined at global and different SDI levels in 2019. ASDR, age-standardized death rate; SDI, sociodemographic index

A total of 111,292 deaths (95% UI, 88,189–132,392) associated with burns were identified globally in 2019, most of which were concentrated in those aged 1–4 years (Fig. 3A). The number of deaths increased from 1990 to 1994, then begins to decline with a slightly fluctuates, especially in males (Fig. 4B, Table 3). The age-standardized deaths rate (ASDR) of burns gradually decreased from 2019 to 1990 (Table 3, Supplemental Figure S 3A, B), and the ASDR and the number of deaths were higher in males than in females, both in 1990 and 2019(Fig. 4D, Table 3, Supplemental Figure S 3E, F). In addition, 13% of deaths were attributable to risk factors for both sexes combined globally in 2019, of which 10% and 3% could be attributed to occupational risks and alcohol use. Besides, death rate distribution by age can be seen in supplemental materials (Fig. 4F, Supplemental Figure S 3D).

**Table 3:** Deaths Cases, Age-Standardized Deaths Rates, and Temporal Trends for Burns From 1990 to 2019

	No. (95% UI)				No. (95% UI)
	1990		2019		1990–2019
Variable	Deaths cases	ASDR per 100,000 people	Deaths cases	ASDR per 100,000 people	EAPC
Global	123,213.46 (95,472.73 to 151,399.41)	2.51 (2.01 to 3)	111,292.39 (88,188.86 to 132,392.34)	1.44 (1.14 to 1.72)	-2.16 (-2.02 to -2.3)
male	66,454.79 (49,765.66 to 74,334.55)	2.84 (2.21 to 3.14)	56,854.07 (44,072.37 to 67,315.99)	1.53 (1.19 to 1.81)	-2.36 (-2.18 to -2.55)
female	56,758.67 (29,388.76 to 78,772.85)	2.23 (1.23 to 3.01)	54,438.32 (39,110.13 to 69,985.43)	1.37 (0.98 to 1.78)	-1.93 (-1.83 to -2.02)
SDI					
High	13,818.45 (13,273.62 to 14,266.95)	1.57 (1.51 to 1.63)	10,257.9 (9298.42 to 11,035.41)	0.68 (0.62 to 0.74)	-3.04 (-2.91 to -3.17)
High-middle	25,586.04 (23,374.97 to 27,776.91)	2.4 (2.19 to 2.6)	21,486.04 (18,615.42 to 23,804.92)	1.23 (1.06 to 1.37)	-2.89 (-2.4 to -3.39)
Middle	34,836.88 (25,141.73 to 43,397.49)	2.38 (1.82 to 2.89)	27,772.69 (20,017.38 to 34,978.77)	1.22 (0.89 to 1.53)	-2.47 (-2.42 to -2.53)
Low-middle	29,736.85 (18,605.80 to 40,630.72)	2.96 (2.02 to 3.88)	28,424.96 (21,859.46 to 34,569.4)	1.84 (1.44 to 2.2)	-1.8 (-1.74 to -1.86)
Low	19,145.18 (12,568.06 to 26,000.12)	4.04 (2.95 to 5.1)	23,254.21 (17,019.13 to 30,275.53)	2.81 (2.08 to 3.55)	-1.26 (-1.23 to -1.29)

*Abbreviations*: *ASDR* Age-standardized deaths rate, *EAPC* Estimated annual percentage change, *SDI* Sociodemographic index, *UI* Uncertainty interval, *CI* Confidence interval

Deaths were lower among all SDI quantiles except for the Low SDI level in 2019 than in 1990, and countries with the Middle SDI level had the highest numbers of deaths compared with countries with other SDI levels in 1990, and Low-middle SDI level in 2019 (Fig. 4C, Table 3). The ASDR increased at all SDI levels, and countries with the Low SDI level had higher ASDR than countries with other SDI levels both in 1990 and 2019 (Supplemental Figure S3B, Table 3). In terms of sex, except for the Low-middle SDI level, the number of deaths was higher in males than females not only in 2019 but also in 1990, surprisingly, the ASDR values distributed mode the same as the distribution of deaths among genders. (Fig. 4D, E, Table 3, Supplemental Figure S3E, F). In 2019, occupational risks are the highest contribution to Deaths in countries with lower SDI levels, while alcohol use is the highest contribution in countries with higher SDI levels (Fig. 4D).

For GBD regions, the numbers of deaths decreased in most of the GBD regions, with the most significant decrease detected in World Bank Upper-Middle Income area (from 44,295.11 [95% UI, 36,096.24–51,049.62] to 32,500.09 [95% UI, 27,061.87–36,865.88]) and Australasia has the lowest deaths in 2019(131.83 [95% UI, 119.11–141.72]), where also has the lowest ASDR in 2019 (0.32 [95% UI, 0.29–0.34]). Asia has the highest death cases in 2019 (57,202.37 [95% UI, 41,804.00–70,564.74]), the most significant increase from 1990 to 2019 was detected in Commonwealth Middle Income area (32,612.48 [95% UI, 19,592.70–45,344.34] to 38,255.11[95% UI, 26,466.17–50,950.82]) (Supplemental Figure S3G). Except for in one GBD region, namely Oceania, the ASDR of burns decreased, where has the highest ASDR in 2019 and has the most significant increased ASDR value among 45 GBD regions (from 6.24 [95% UI, 1.65–3.10] to 6.34 [95% UI, 1.17 -10.61]). The most significant decrease in ASDR was detected in Central Asia (from 5.66 [95% UI, 5.37–6.03] to 2.64 [95% UI, 2.28–3.10]) (Supplemental Figure S 3H).

**Fig. 5:**
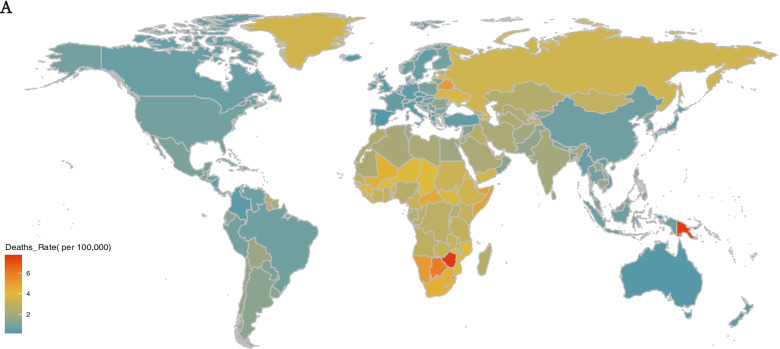
The map of mortality rate in 2019. **A** the map of ASDR in 2019 among 204 countries and territories

At the national level, the highest number of deaths was recorded in India in 2019 (25,876.39 [95% UI, 16,992.26–37,389.59]), an increase of 12% from 1990 (23,031.84 [95% UI, 12,259.04–33,464.85]). The deaths number of India increased the most globally, accounting for about 23% of global deaths in 2019, 27% and 73% of which occurred in males and females, respectively. This was followed by deaths in the Mainland of China (11,095.91 [95% UI, 7,938.48–14,085.43]), 63% and 37% of which occurred in males and females, respectively. In India, 4.8% of age-standardized deaths result from occupational injuries, and deaths peaked in those 20–24 years of age in 2019. The most significant decrease in the number of deaths was detected in Mainland of China (from 17,289.45 [95% UI, 12,545.78–21,015.43] to 11,095.91 [95% UI, 7,938.48–14,085.43]) (Supplemental Figure S3I), Tokelau has the lowest death number and almost reach zero(0.01 [95% UI, 0.01–0.02]) in 2019. Lesotho had the highest ASDR (7.75 [95% UI, 5.63–10.45]), whereas Singapore had the lowest (0.19 [95% UI, 0.17–0.20]) in 2019. The most significant increase in the ASDR was detected in Lesotho (from 6.19 [95% UI, 4.38–8.82] to 7.75 [95% UI, 5.63–10.45]), whereas the most significant decrease was detected in Armenia (from 7.38 [95% UI, 6.89–7.91] to 1.28 [95% UI, 1.09–1.49]) (Supplemental Figure S3J). Furthermore, we visualized the number of deaths in 2019 among 204 countries and territories by map (Fig. 5A).

#### Temporal trends of burns

We analyzed the temporal trends in burns incidence, DALYs, and deaths at the national, regional, and global levels from 1990 to 2019. Incidence, DALYs, and deaths of burns cline to decrease at the global level and all SDI levels. Meanwhile, the EAPC of incidence and deaths are all higher at the High SDI level, and the EAPC of DALYs a higher at the high-middle SDI level.

**Fig. 6:**
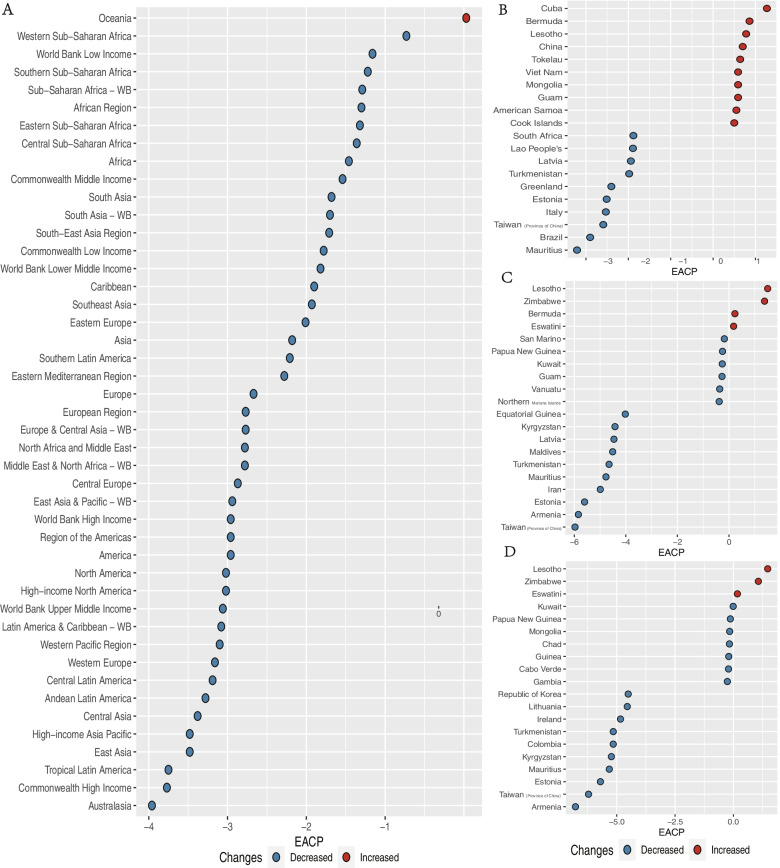
Temporal Trends of burns. **A** the EAPC of death in 45 GBD regions. **B** the top positive and negative EAPC of incidence among 204 countries and territories. **C** the top positive and negative EAPC of DALYs among 204 countries and territories. **D** the top positive and negative EAPC of Deaths among 204 countries and territories. GBD, Global Burden of Disease; EAPC, estimated annual percentage change

In the 45 GBD regions, except for four regions, namely East Asia, Western Pacific Region, East Asia & Pacific – WB, Oceania, the EAPCs of incidence were negative (Fig. 6A); except for one region, namely Oceania, the EAPC of DALYs and deaths were negative, suggesting that the incidence, DALYs and deaths of burns was decreasing over time in most of the GBD regions (Supplemental Figure S 4A, B).

**Fig. 7:**
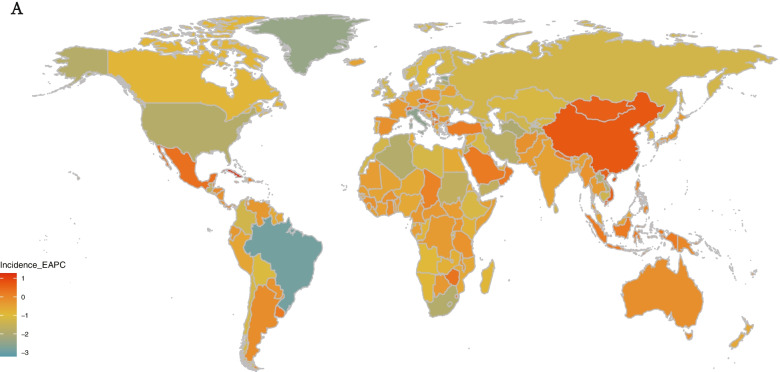
The map of Incidence EAPC. **A** the map of Incidence EAPC among 204 countries and territories. EAPC, estimated annual percentage change

At the national level, most of EAPCs were negative, the highest positive EAPCs of incidence, DALYs, and deaths were observed in Cuba, Lesotho, and Lesotho, respectively; whereas the highest negative EAPCs of incidence, DALYs, and deaths were observed in Mauritius, Taiwan (Province of China) and Armenia, respectively (Fig. 6B, C, D). Furthermore, we visualized the EAPCs of incidence, DALYs, and deaths in 2019 among 204 countries and territories by map (Fig. 7A, Supplemental Figure S 4C, D).

#### Relationship of EAPC of burns incidence, DALYs, and death with SDI, UHC, and GDP.

**Fig. 8:**
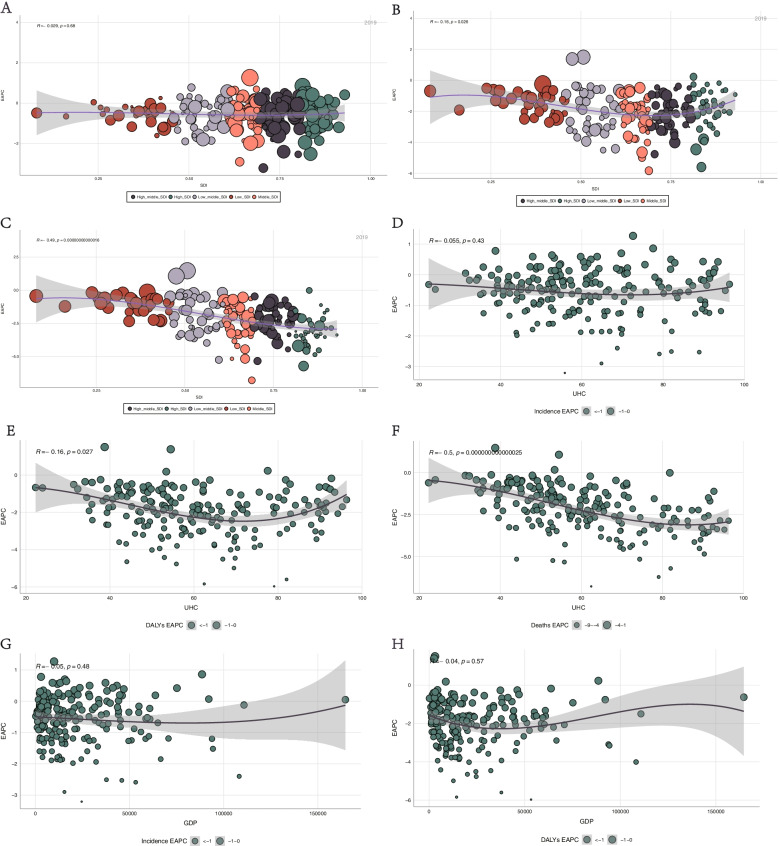
Relationship of EAPCs in burns incidence, DALYs, and death with SDI, UHC, and GDP. **A** correlation analysis of the EAPC of ASIR with SDI. **B** correlation analysis of the EAPC of ASDAR with SDI. **C** correlation analysis of the EAPC of ASDR with SDI. **D** correlation analysis of the EAPC of ASIR with UHC. **E** correlation analysis of the EAPC of ASDAR with UHC. **F** correlation analysis of the EAPC of ASDR with UHC. **G** correlation analysis of the EAPC of ASIR with GDP. **H** correlation analysis of the EAPC of ASDAR with GDP. EAPC, estimated annual percentage change; DALYs, disability-adjusted life years; SDI, sociodemographic index; UHC, universal health coverage; GDP, gross domestic product; ASIR, age-standardized incidence rate; ASDAR, age-standardized DALYs rate

We analyzed the correlation between the 2019 SDI and EAPCs in burns incidence, DALYs, and deaths. SDI was negatively correlated with all EAPCs (incidence, *R* =  − 0.029, p = 0.68; DALYs, R =  − 0.16, *p* = 0.026; deaths, *R* =  − 0.49, *p* = 0.00000000000016), indicating that incidence, DALYs, and deaths of burns declined with increasing SDI values. SDI values also negatively and significantly correlated with the EAPC of DALYs and deaths, meaning that the SDI value had an impact on temporal trends related to DALYs and deaths of burns (Fig. 8A, B, C).

Achieving universal health coverage (UHC) involves all individuals receiving the health services they need, of high quality, without experiencing financial difficulty and the UHC effective coverage index provides the understanding of whether health services are aligned with countries' health profiles and are of sufficient quality to produce health gains for populations [21]. To better understand the distribution of burns based on healthcare system performances of countries, we examined the relationship between EAPCs in burns incidence, DALYs, and deaths with the UHC index by Pearson correlation analysis. The SDI was negatively correlated with all EAPC measurements (incidence, *R* =  − 0.055, *p* = 0.43; DALYs, *R* =  − 0.16, *p* = 0.027; deaths, *R* =  − 0.5, *p* = 0.000000000000025). These results suggest that the UHC index has a vital impact on temporal trends related to DALYs and deaths of burns (Fig. 8D, E, F).

Spending on healthcare varied within and across income groups and geographical regions, which is expected to rise [23]. We examined the relationship between EAPCs in burns incidence, DALYs, and deaths with GDP by Pearson correlation analysis. GDP was negatively correlated with all EAPC measurements (incidence, *R* =  − 0.05, *p* = 0.48; DALYs, *R* =  − 0.04, *p* = 0.57; deaths, *R* =  − 0.37, *p* = 0.000000041). These results suggest that GDP has a vital impact on the temporal trend of deaths (Fig. 8G, H, Supplemental Figure S 5).

## Discussion

Burn injuries are under-appreciated trauma that is an important cause of morbidity and mortality in many parts of the world [4, 24, 25]. To our knowledge, this study is the first to comprehensively analyze international burn trends in 204 countries and territories from 1990 to 2019. The study improved our understanding of the global distribution and burden of burns.

Several previous works indicated that burn injuries are decreasing, which happens especially in high-income countries, however, the prevalence of burn injuries remains high elsewhere with lower income, but those research mainly operated on the national level and incomes level [5, 26–31]. First, combining the results of previous research and our study, we can conclude that the age-standardized rates of burn incidence, DALYs, and mortality will continuously decrease, but the number of new burn cases has an increasing tendency globally. Although the causes of burn injuries in children and adolescents in Eastern and Western countries are similar [32–34], burns were more prevalent in younger age groups [33–36], which is possible because children have a low ability to avoid risks, unable to verbalize their needs, have different airway anatomy than adults, resulting in a higher incidence of upper airway obstruction due to edema and are prone to the development of hypothermia [37–40].

Further, we analyzed the burns epidemiological indicators on SDI level, the result shows that burns lead to a persistent healthcare burden on each country, especially those with lower SDI levels. SDI index contains per capita income, average years of education, and total fertility rates, which increased the analysis angle of socio-demographic aspects affects on burns prevalence than previous works [28, 34, 41]. In addition, the correlation of EAPCs with SDI, UHC, and GDP indicates that prevention burns not only depend on health spending per capita but also depend on the education level per capita and healthcare system performance, but it does not mean higher health spending corresponds to higher UHC index, which needs high efficiency of translating health spending into individuals health gains [21, 42].

There are a lot of risk factors for burns, the aspects including individual, family, and society [28], we evaluated a set of risks for burns, and we can find that although these countries with lower SDI occupied a high-level occupational risk, it remains has great influence in many countries. what's surprising is that alcohol use is a quite effective risk factor for burns. At the national level, the highest ASIR highlights the substantial societal burden and disease management challenges that burns continue to pose, while a substantial decrease in the ASIR suggests successful prevention and healthcare policies that could be used as a reference point for establishing new policies elsewhere.

### Limitations

This study suffers from the general limitations of GBD studies. First, the accuracy of GBD estimation depends largely on the quality and quantity of data used since there are under-reporting and under-diagnosis during burns registration. Second, death certification accuracy has international variability and the co-morbidities that are often associated with burns can add further ambiguity when identifying the true cause of death. Third, the data could not be explored further to extract information related to severity, and treatments, as such information was not provided in the GHDx. Therefore, we could not generate a detailed etiological understanding of global changes in burn patterns. Lastly, due to the observational nature of this analysis, there are likely to be a number of unmeasured confounding factors not discussed and causal statements about the trends observed cannot be made.

## Conclusions

Globally, the age-standardized rates of burn incidence, DALYs, and mortality, as well as the number of burn DALYs and death cases will continuously decrease, but the number of new burn cases has an increasing tendency globally. In addition, the EAPCs of burns in incidence, DALYs, and deaths indicated that the burden of burns was considered to be decreasing in most of the regions. And from the relationship of EAPCs with SDI, UHC index, and GDP, indicate that prevention burns not only depend on health spending per capita but also depend on the education level per capita and healthcare system performance, but it does not mean higher health spending corresponds to higher UHC index, which needs high efficiency of translating health spending into individuals health gains.

## Supplementary Information

**Additional file 1: Supplemental Figure S1.** Incidence of burns. A, the global changing trend in ASIR by sex from 1990 to 2019. B, a comparison of the number of new cases between 1990 and 2019 at global and different SDI levels. C, distribution of new cases among different age categories in 1990. D, distribution of incidence rate among different age categories in 2019. E, distribution of incidence rate among different age categories in 1990. F, a comparison of the number of new cases by sex globally and at different SDI levels in 2019. G, a comparison of the number of new cases by sex at global and different SDI levels in 1990. H, a comparison of the ASIR by sex at global and different SDI levels in 1990. I, the range of change in the number of new cases by sex in 2019 compared with 1990 in 45 GBD regions. J, the rangeability in ASIR by sex in 2019 compared with 1990 in 45 GBD regions. K, the map of ASIR in 2019 among 204 countries and territories. SDI, sociodemographic index; ASIR, age-standardized incidence rate; GBD, Global Burden of Disease.

**Additional file 2: Supplemental Figure S2.** DALYs of burns. A, the global changing trend in the number of ASDAR by sex from 1990 to 2019. B, a comparison of the ASDAR between 1990 and 2019 at global and different SDI levels. C, distribution of DALYs rate among different age categories in 2019. D, distribution of DALYs rate among different age categories in 1990. E, the range of change in DALYs by sex in 2019 compared to 1990 in 45 GBD regions. F, the range of change in ASDAR by sex in 2019 compared to 1990 in 45 GBD regions. G, the top increased or decreased in DALYs by sex in 2019 compared with 1990 among 204 countries and territories. H, the top increased or decreased in ASDAR by sex in 2019 compared with 1990 among 204 countries and territories. I, distribution of DALYs among different age categories in 1990. J, the map of DALYs in 2019 among 204 countries and territories. K, the map of ASDAR in 2019 among 204 countries and territories. DALYs, disability-adjusted life years; SDI, sociodemographic index; ASDAR, age-standardized DALYs rate; GBD, Global Burden of Disease.

**Additional file 3: Supplemental Figure S3.** Mortality of burns. A, the global changing trend of the ASDR by sex from 1990 to 2019. B, a comparison of ASDR between 1990 and 2019 at global and between different SDI levels. C, distribution of death rate among different age categories in 2019. D, distribution of the number of death cases among different age categories in 1990. E, a comparison of the number of death cases by sex at global and different SDI levels in 1990. F, a comparison of ASDR by sex at global and between different SDI levels in 2019. G, the range of change in the number of death cases by sex in 2019 compared with 1990 in 45 GBD regions. H, the range of change in ASDR by sex in 2019 compared with 1990 in 45 GBD regions. I, the top increased or decreased in the number of death cases by sex in 2019 compared with 1990 among 204 countries and territories. J, the top increased or decreased in ASDR by sex in 2019 compared with 1990 among 204 countries and territories. K, the map of death cases number in 2019 among 204 countries and territories. SDI, sociodemographic index; ASDR, age-standardized death rate; GBD, Global Burden of Disease.

**Additional file 4: Supplemental Figure S4.** Temporal Trends of burns. A, the EAPC of incidence in 45 GBD regions. B, the EAPC of deaths in 45 GBD regions. C, the map of deaths EAPC in 2019 among 204 countries and territories. D, the map of DALYs EAPC in 2019 among 204 countries and territories. EAPC, estimated annual percentage change; DALYs, disability-adjusted life years; GBD, Global Burden of Disease.

**Additional file 5: Supplemental Figure S5.** Relationship of EAPCs in burns incidence, DALYs, and death with SDI, UHC, and GDP. A, correlation analysis of the EAPC of ASDR with GDP. EAPC, estimated annual percentage change; DALYs, disability-adjusted life years; SDI, sociodemographic index; UHC, universal health coverage; GDP, gross domestic product; ASDR, age-standardized death rate.

**Additional file 6: Table S3.** GBD location hierarchy with levels (*Table from:Diseases, G.B.D. and C. Injuries, Global burden of 369 diseases and injuries in 204 countries and territories, 1990-2019: a systematic analysis for the Global Burden of Disease Study 2019. Lancet (London, England), 2020. 396(10258): p. 1204-1222.* ).

## Data Availability

The datasets generated and/or analyzed during the current study are available in the GBD 2019 repository, which is freely available on http://ghdx.healthdata.org/gbd-results-tool.
