# Supplementary figures and images for: The epidemiological characteristic and trends of burns globally
Source: BMC Public Health. 2022 Aug 22;22:1596. doi: 10.1186/s12889-022-13887-2 (PMC9396832; doi:10.1186/s12889-022-13887-2)

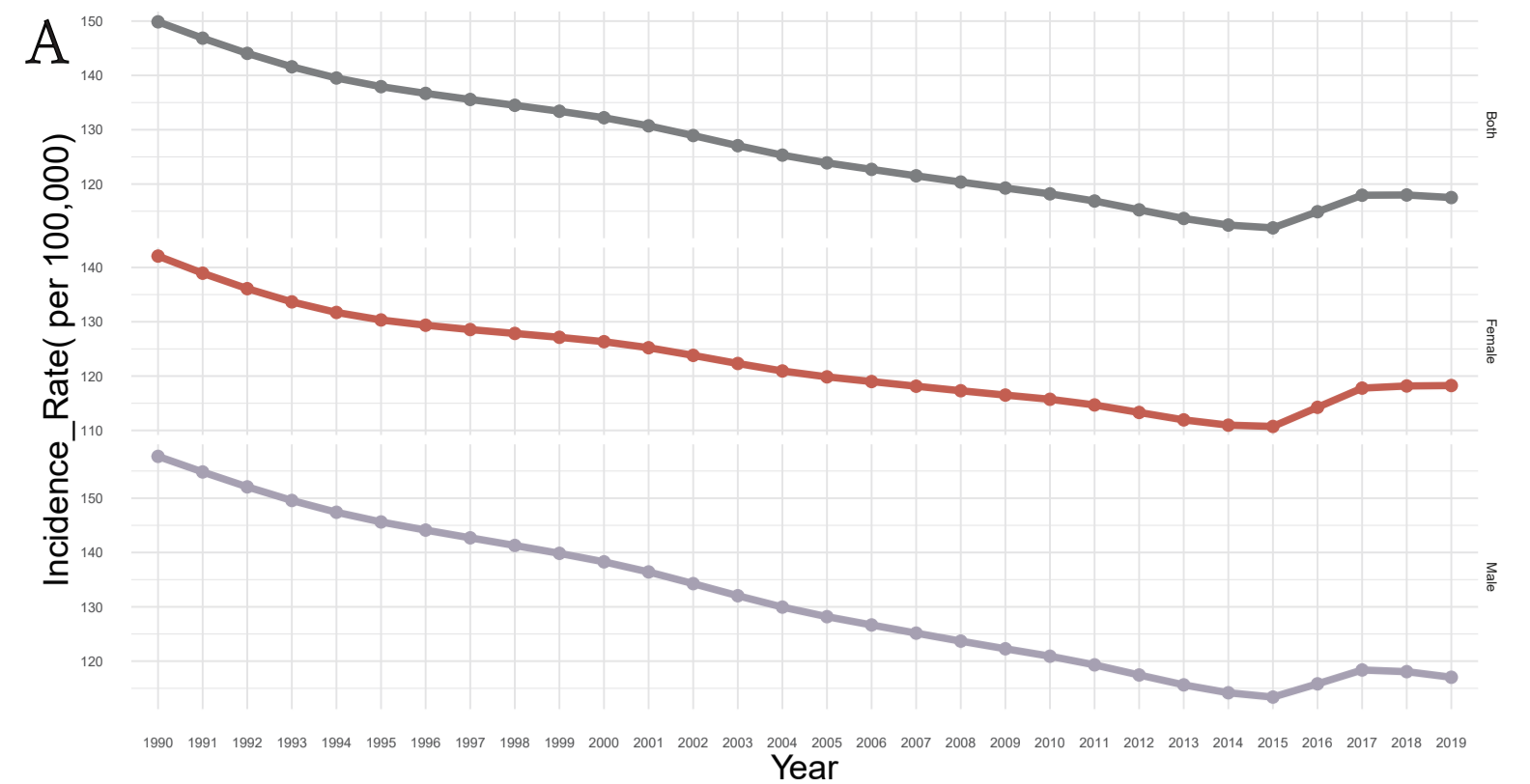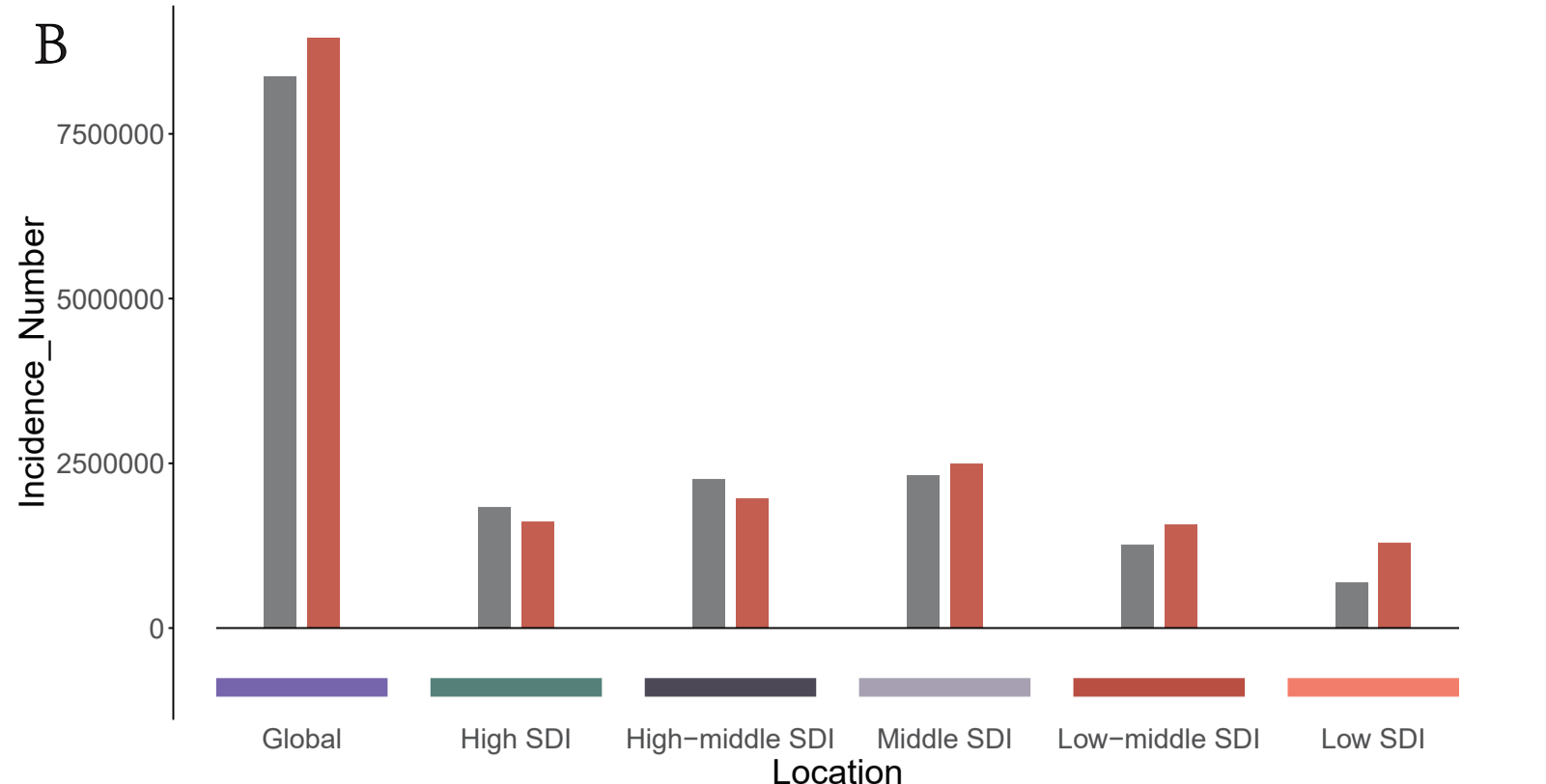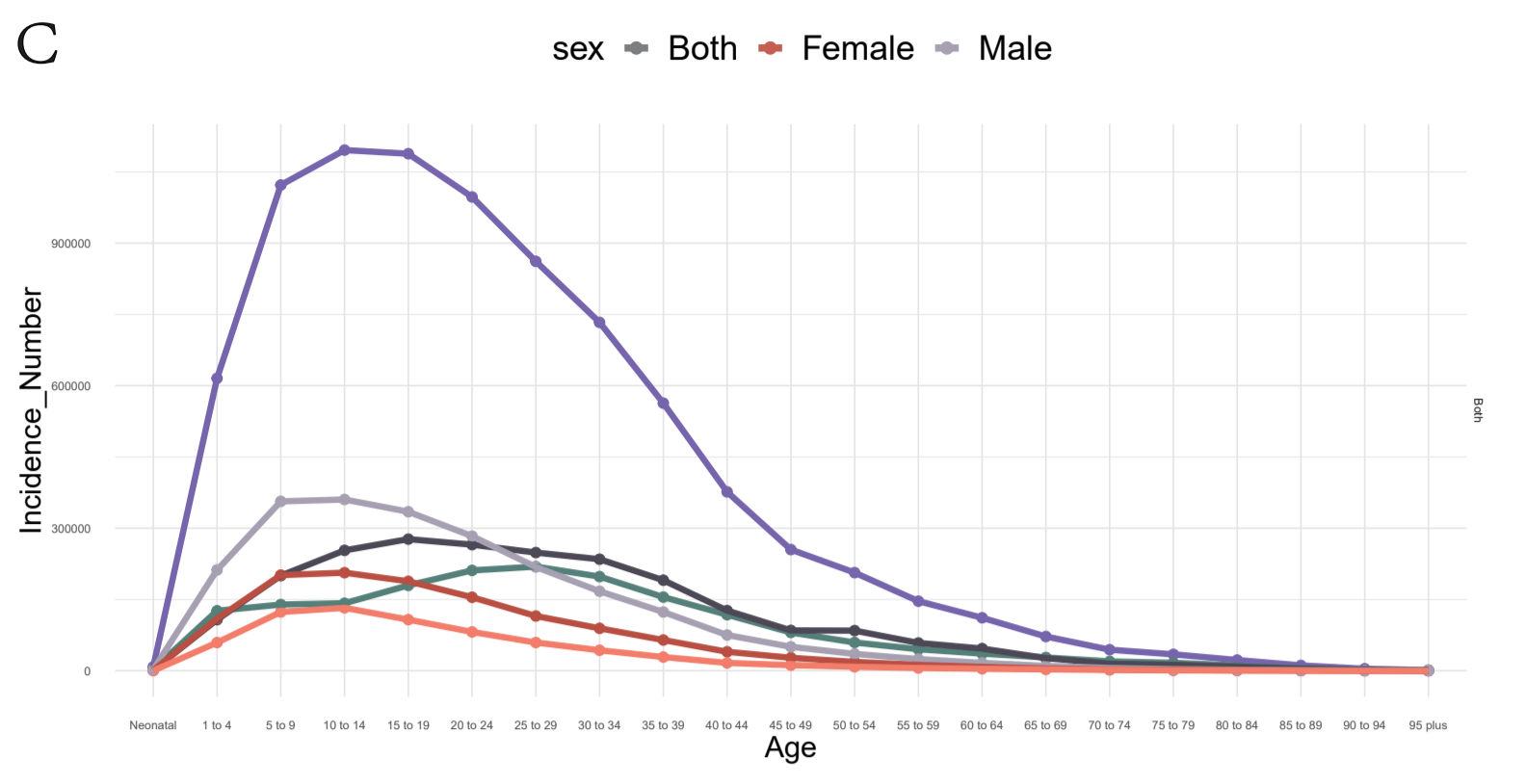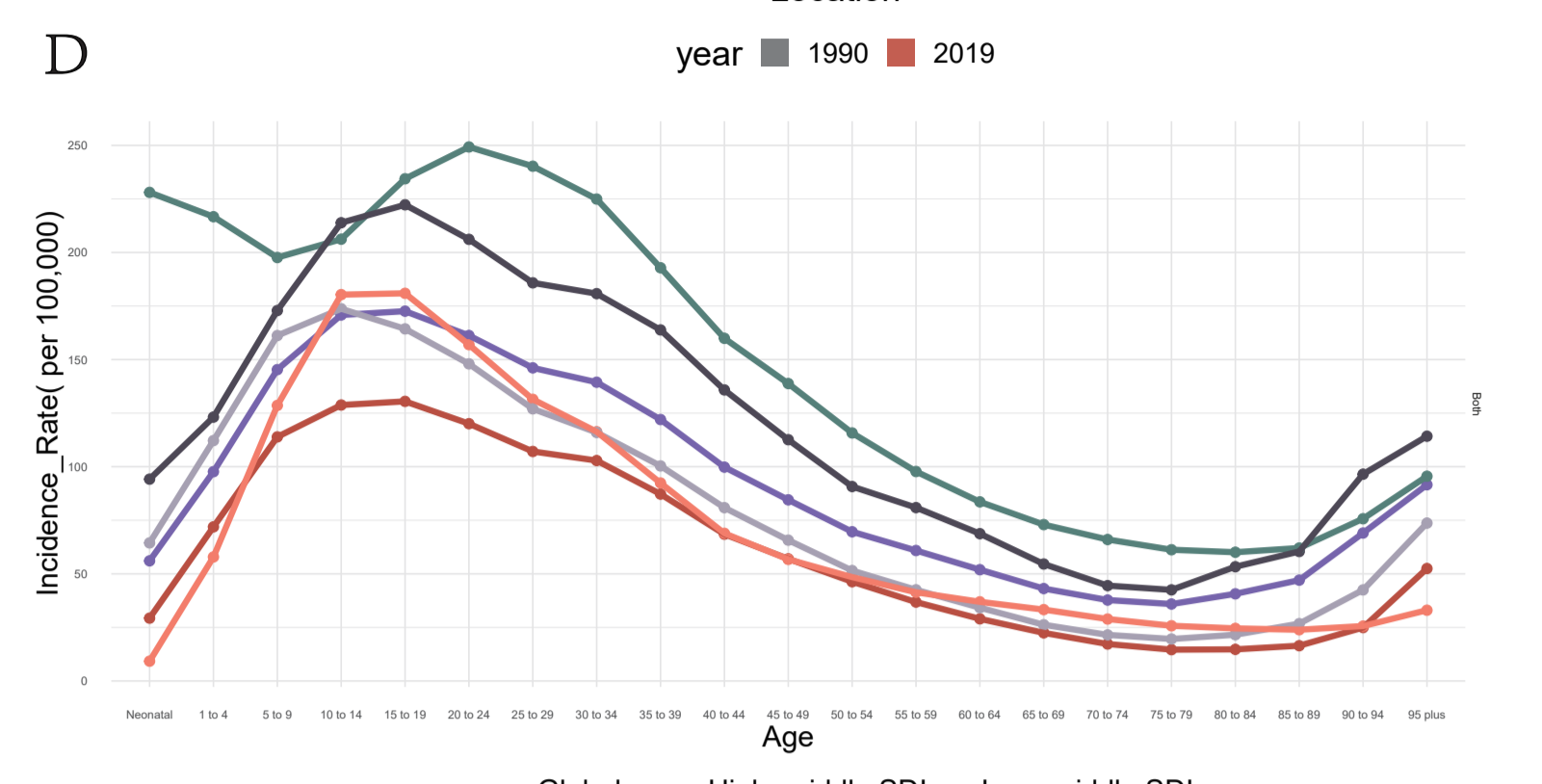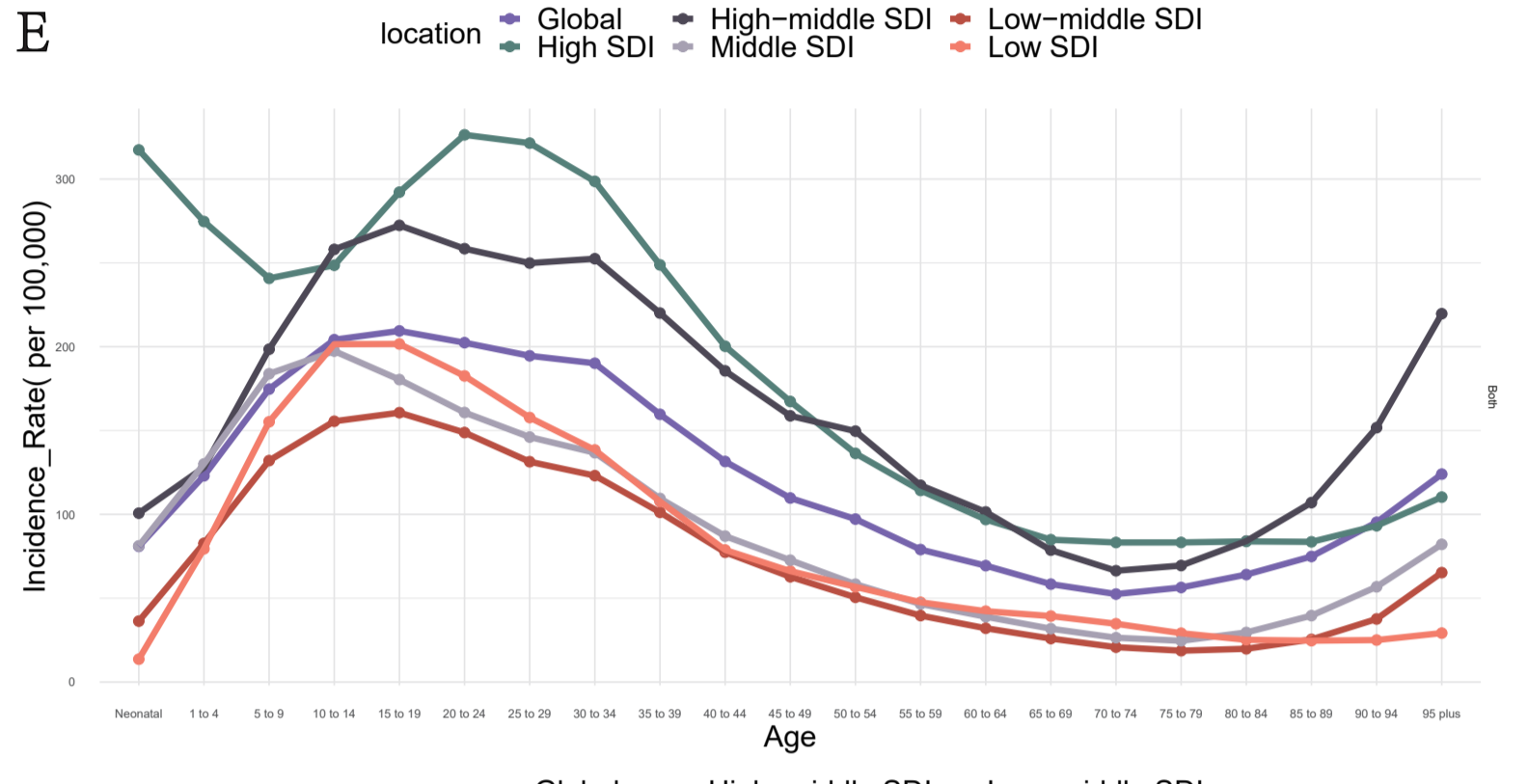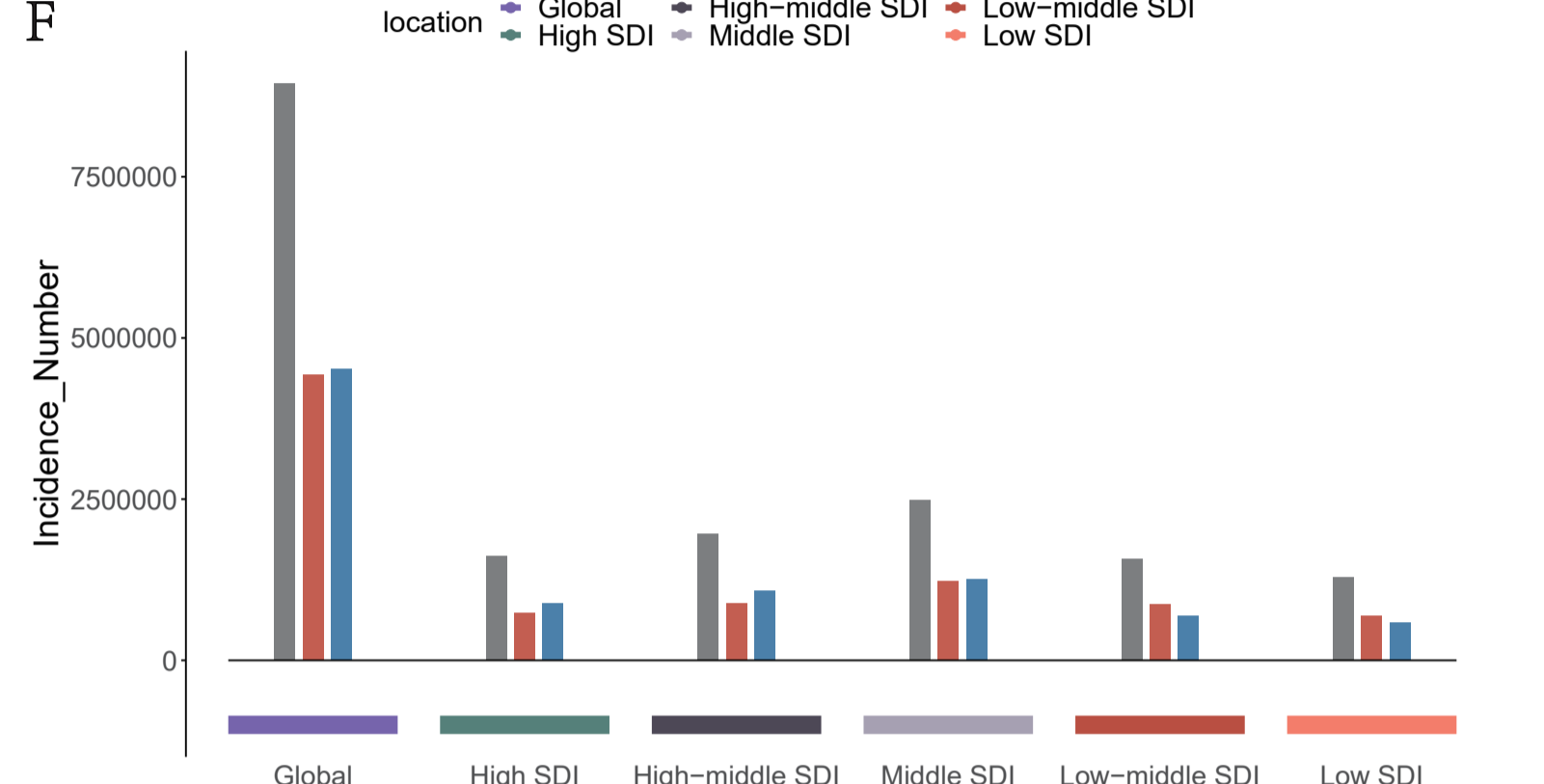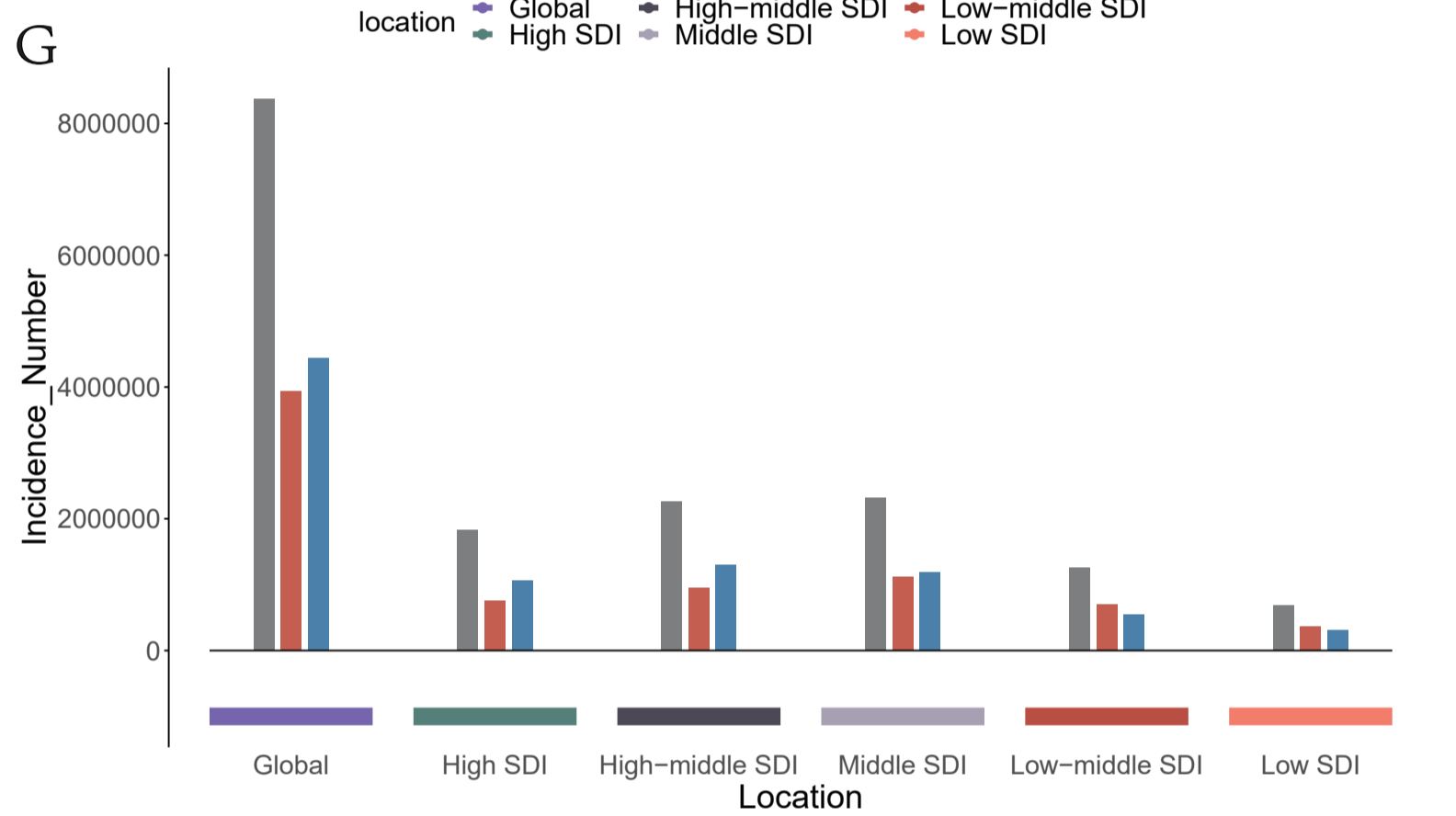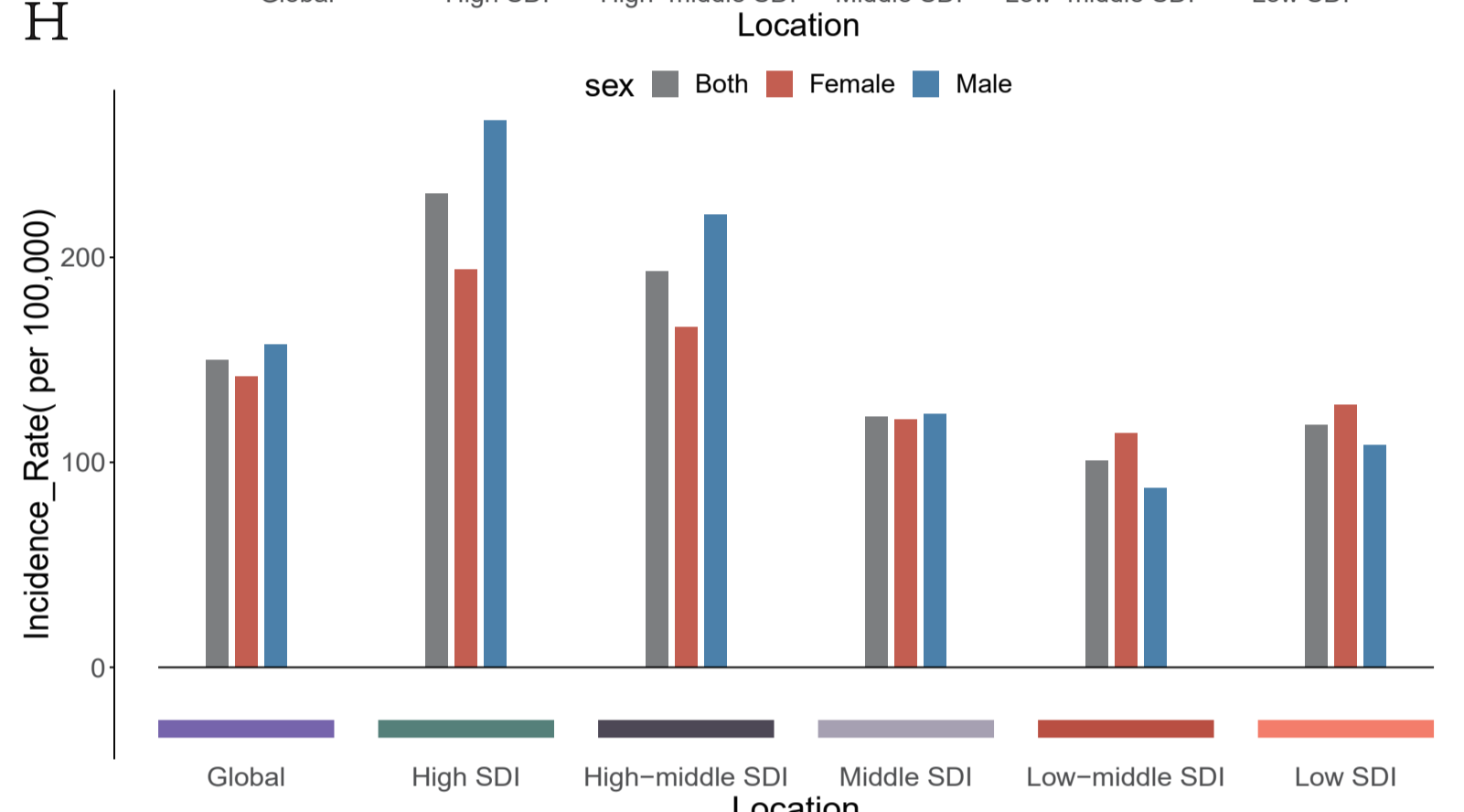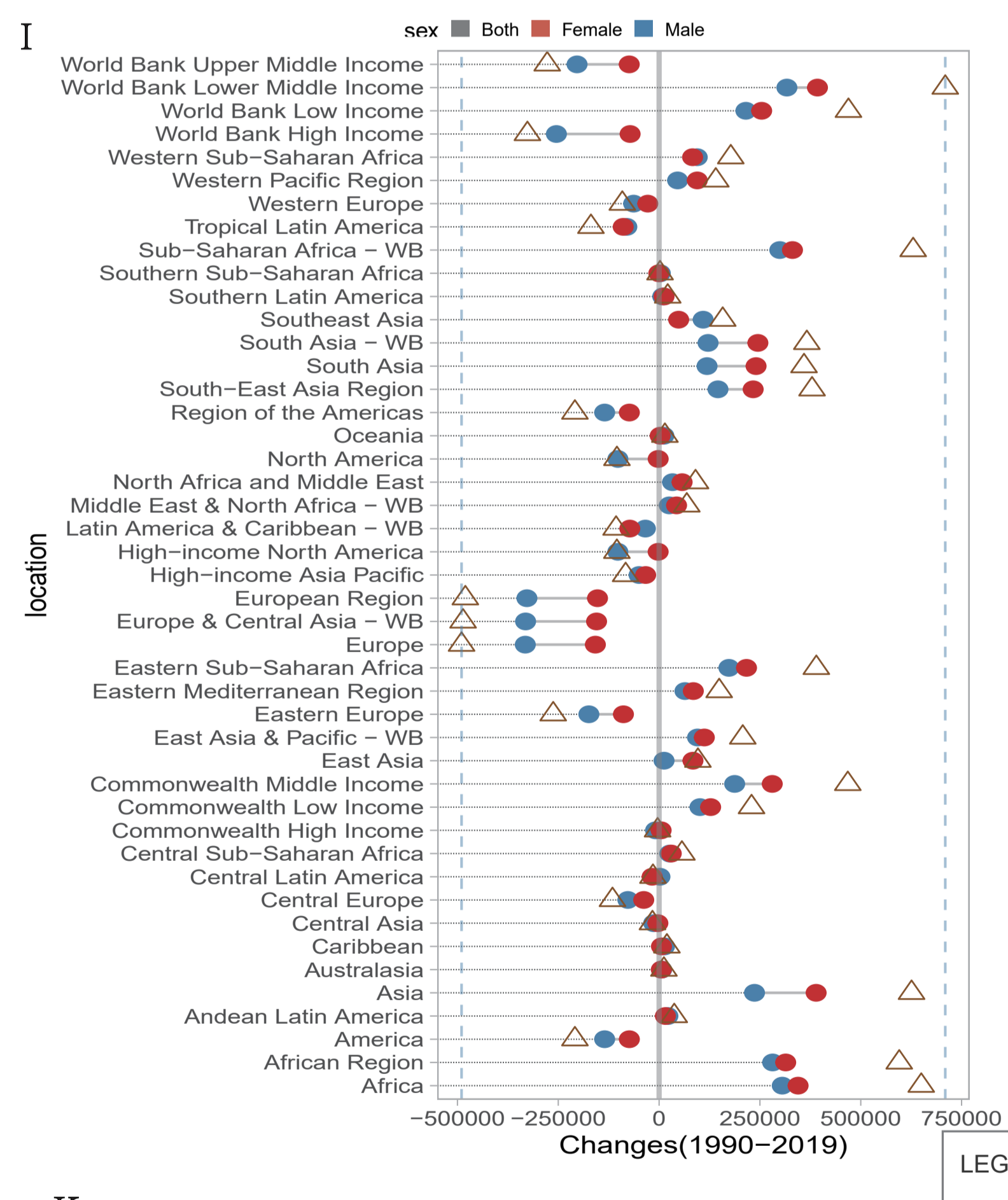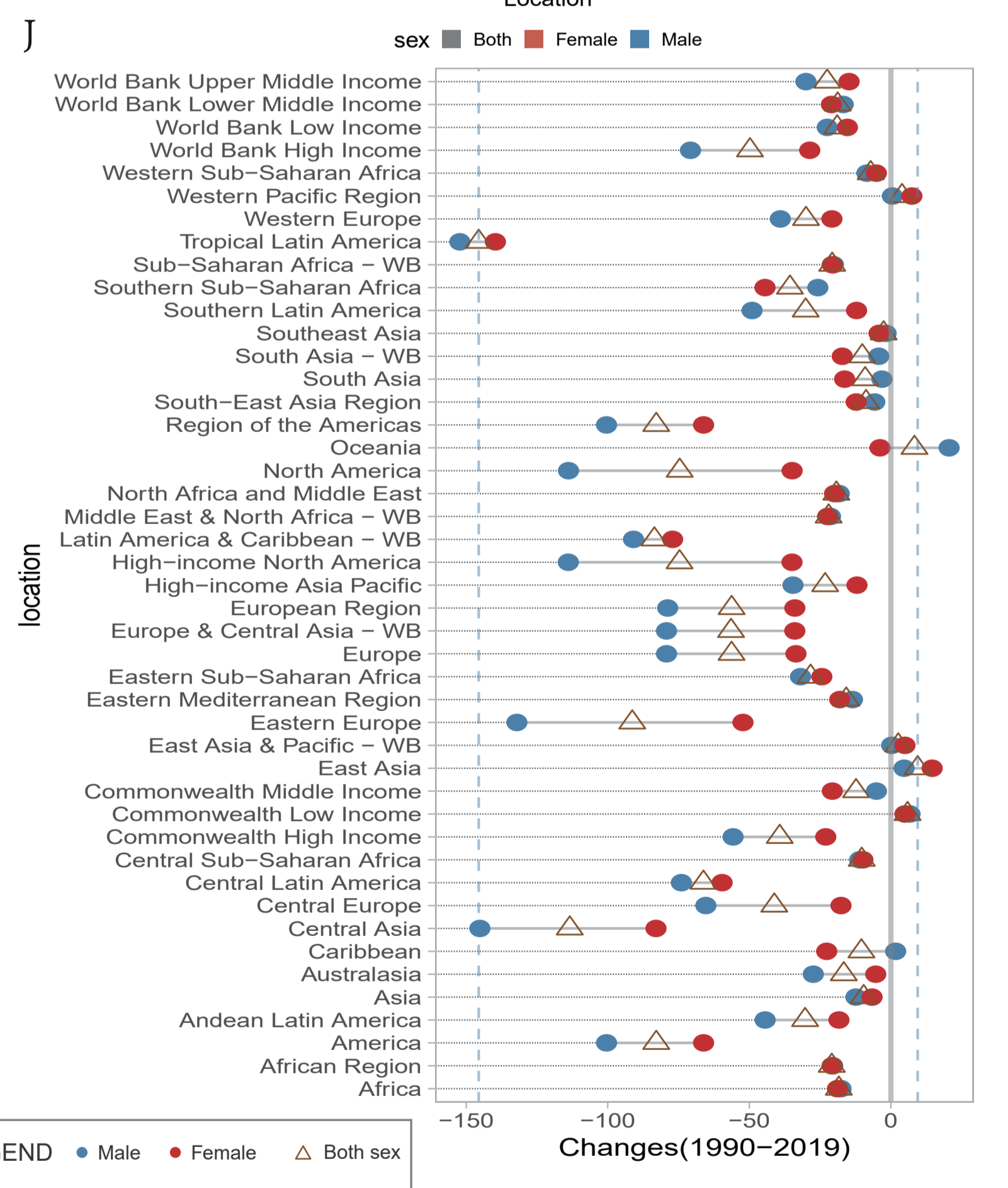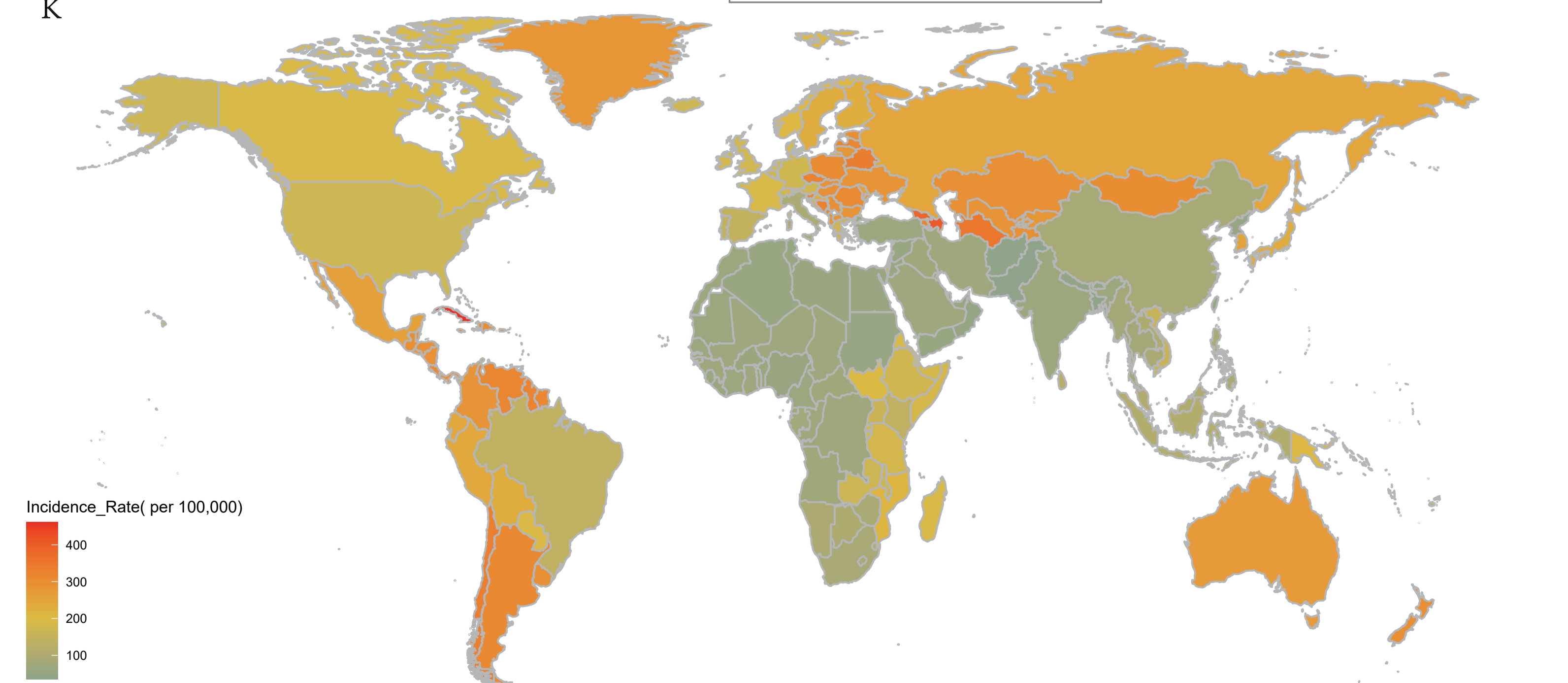

Supplement: Supplementary file 1 — Additional file 1: Supplemental Figure S1. Incidence of burns. A, the global changing trend in ASIR by sex from 1990 to 2019. B, a comparison of the number of new cases between 1990 and 2019 at global and different SDI levels. C, distribution of new cases among different age categories in 1990. D, distribution of incidence rate among different age categories in 2019. E, distribution of incidence rate among different age categories in 1990. F, a comparison of the number of new cases by sex globally and at different SDI levels in 2019. G, a comparison of the number of new cases by sex at global and different SDI levels in 1990. H, a comparison of the ASIR by sex at global and different SDI levels in 1990. I, the range of change in the number of new cases by sex in 2019 compared with 1990 in 45 GBD regions. J, the rangeability in ASIR by sex in 2019 compared with 1990 in 45 GBD regions. K, the map of ASIR in 2019 among 204 countries and territories. SDI, sociodemographic index; ASIR, age-standardized incidence rate; GBD, Global Burden of Disease. [file 12889_2022_13887_MOESM1_ESM.pdf]

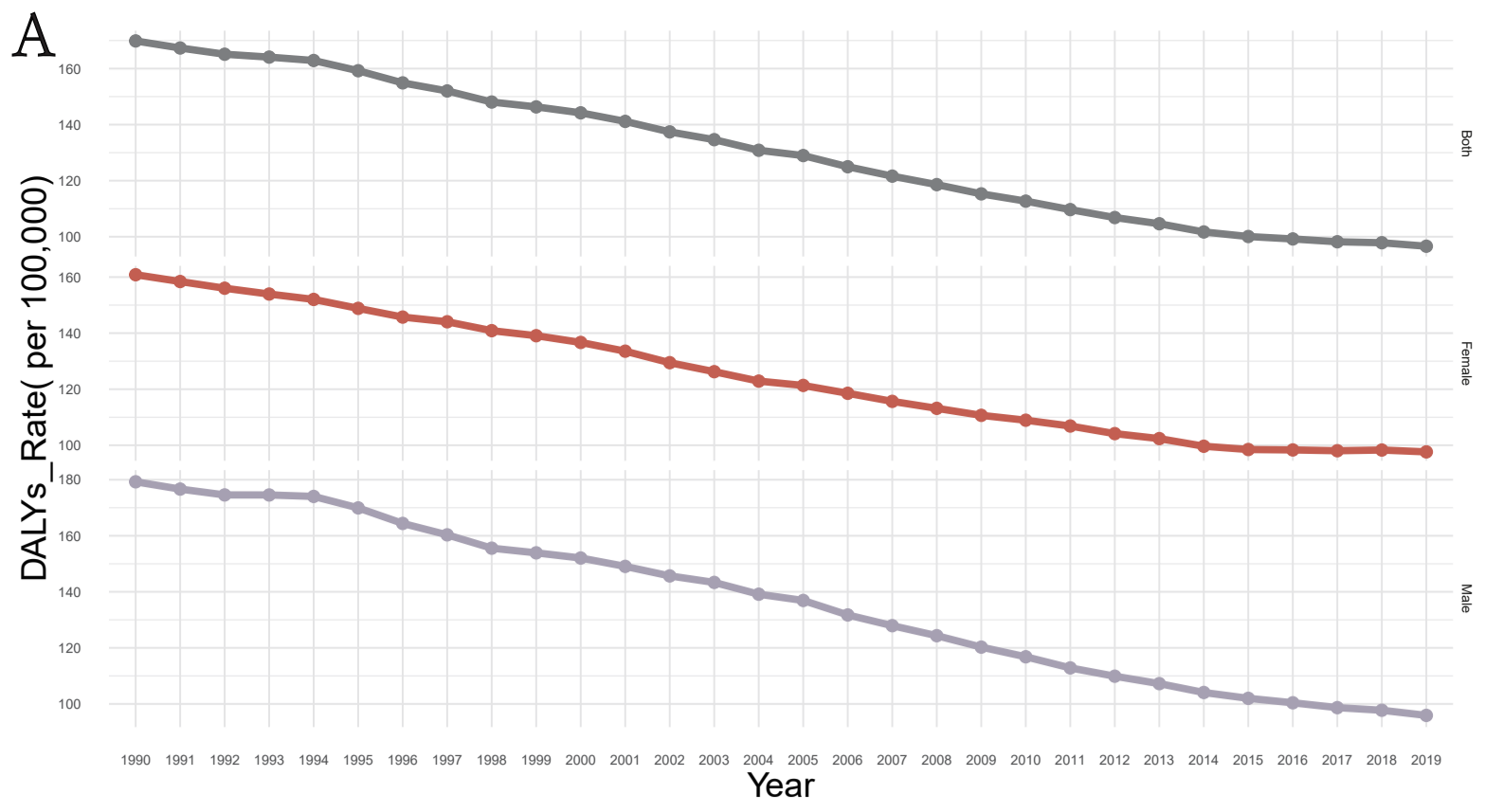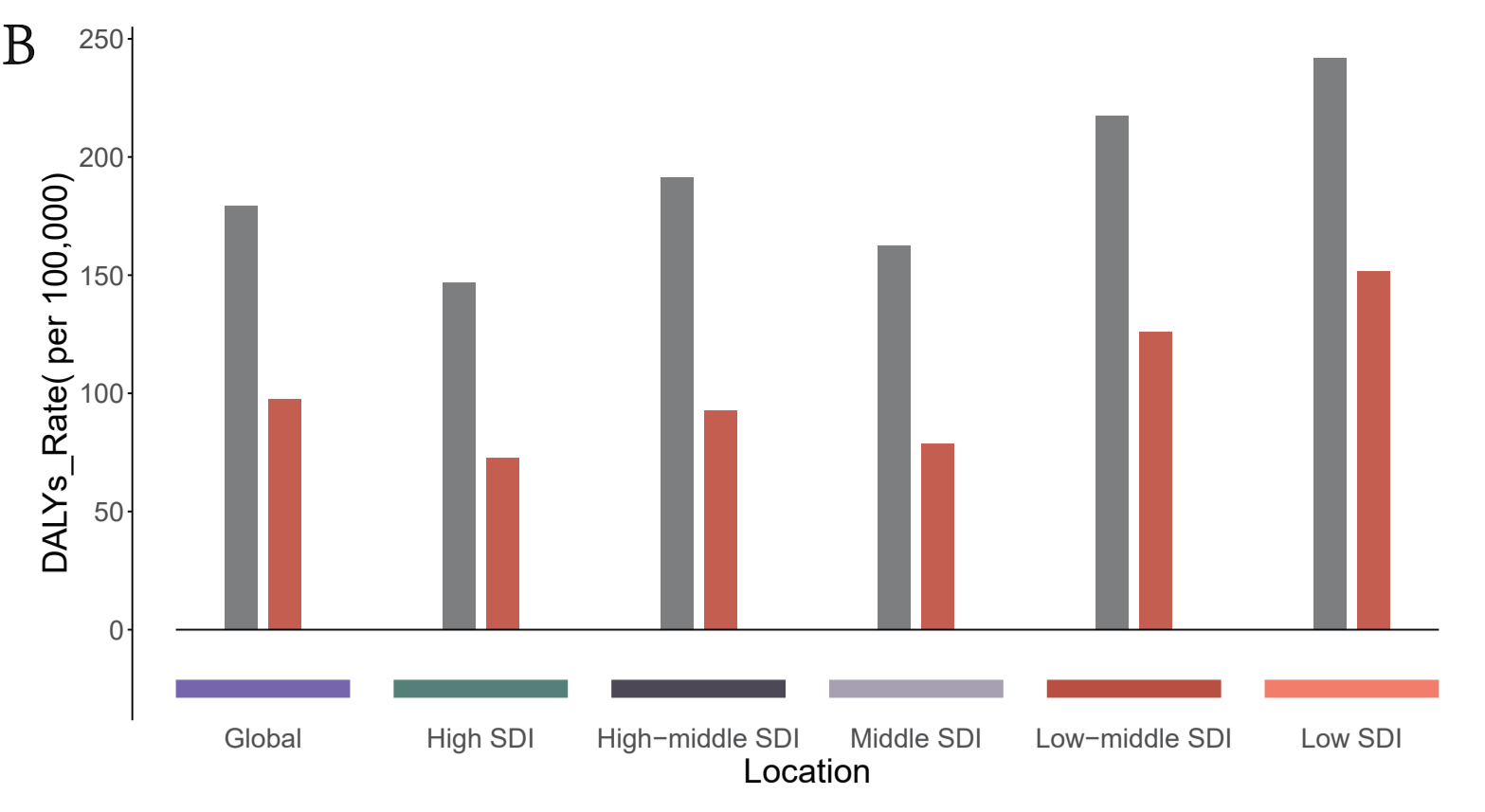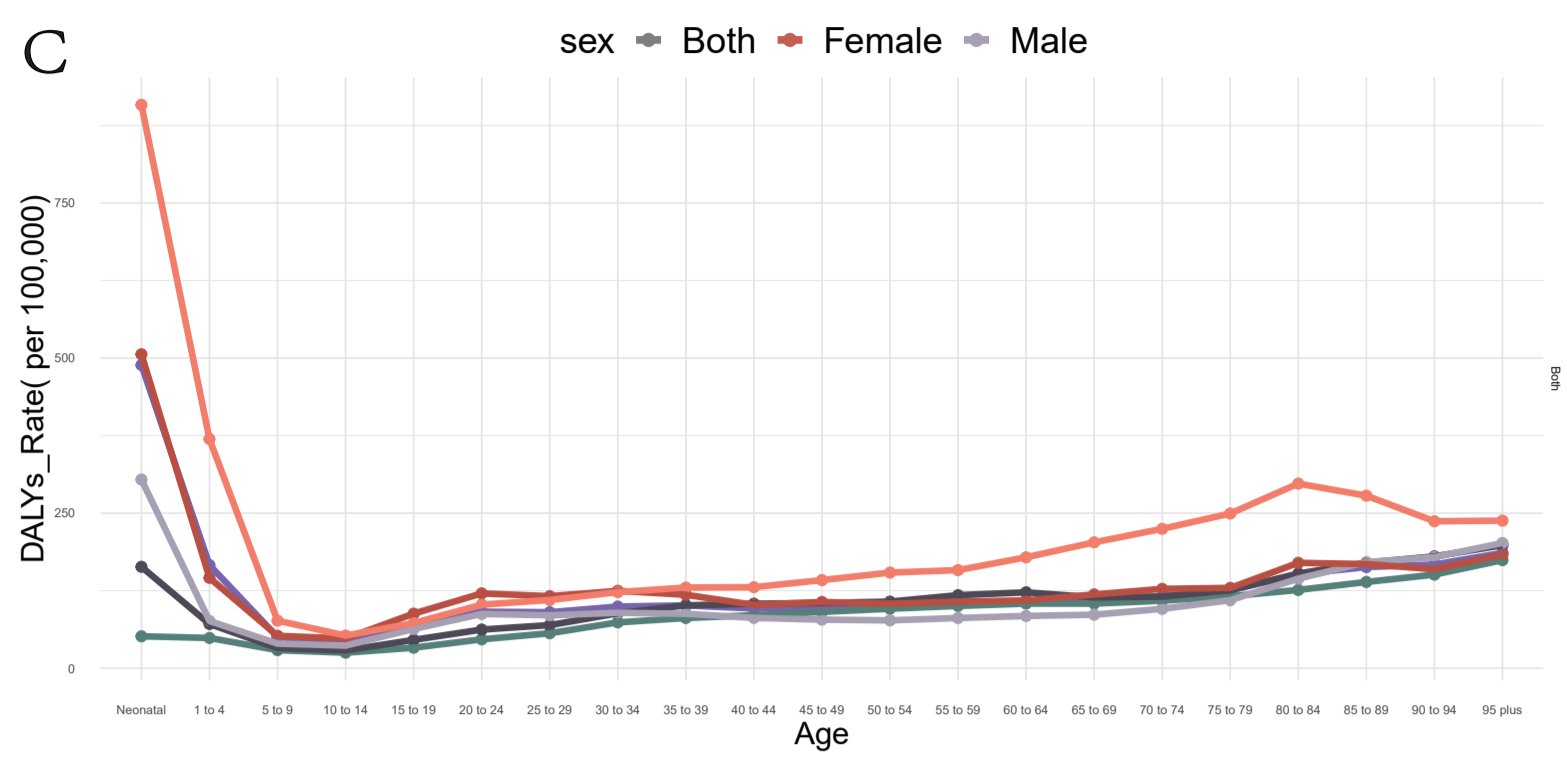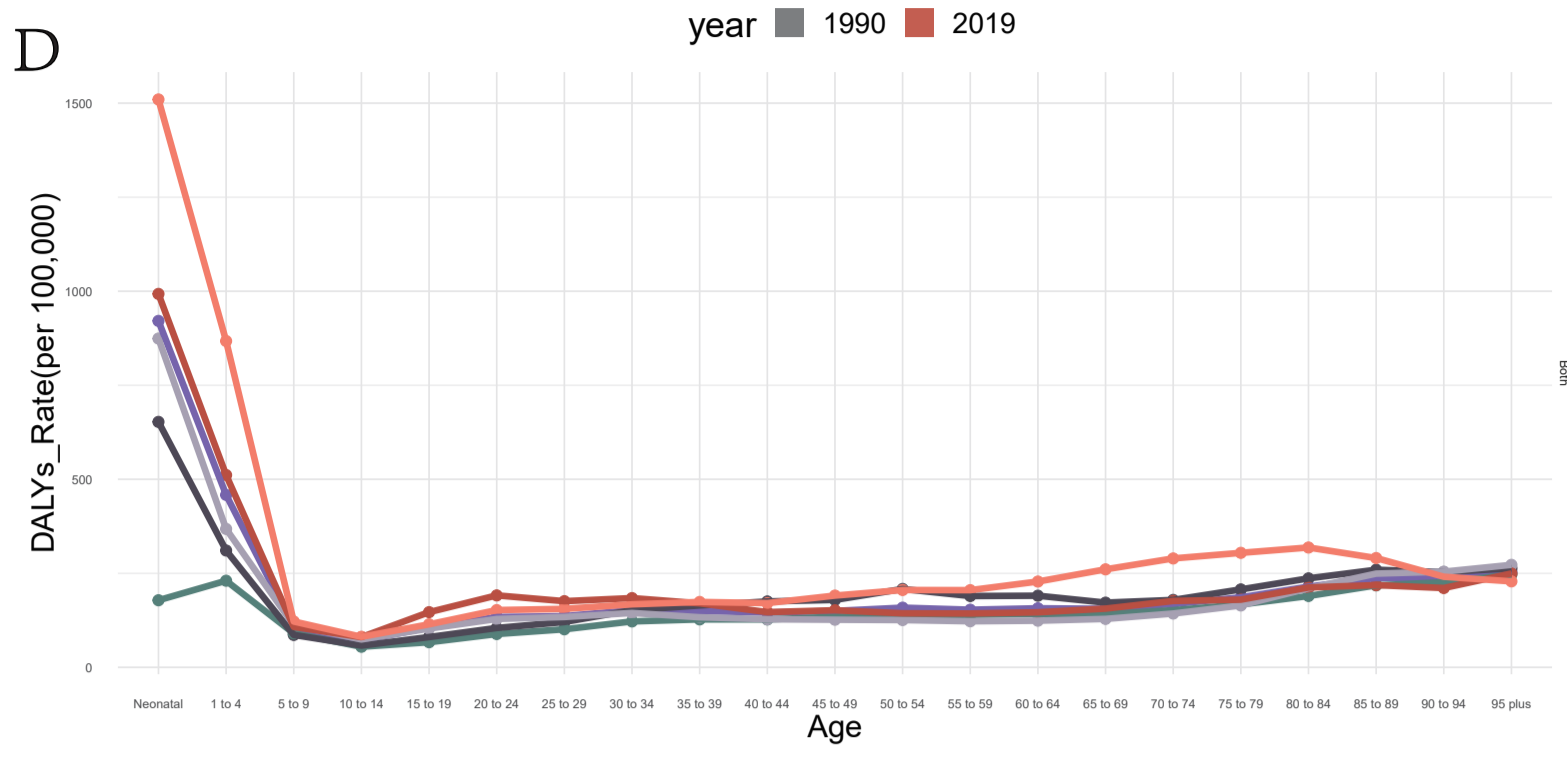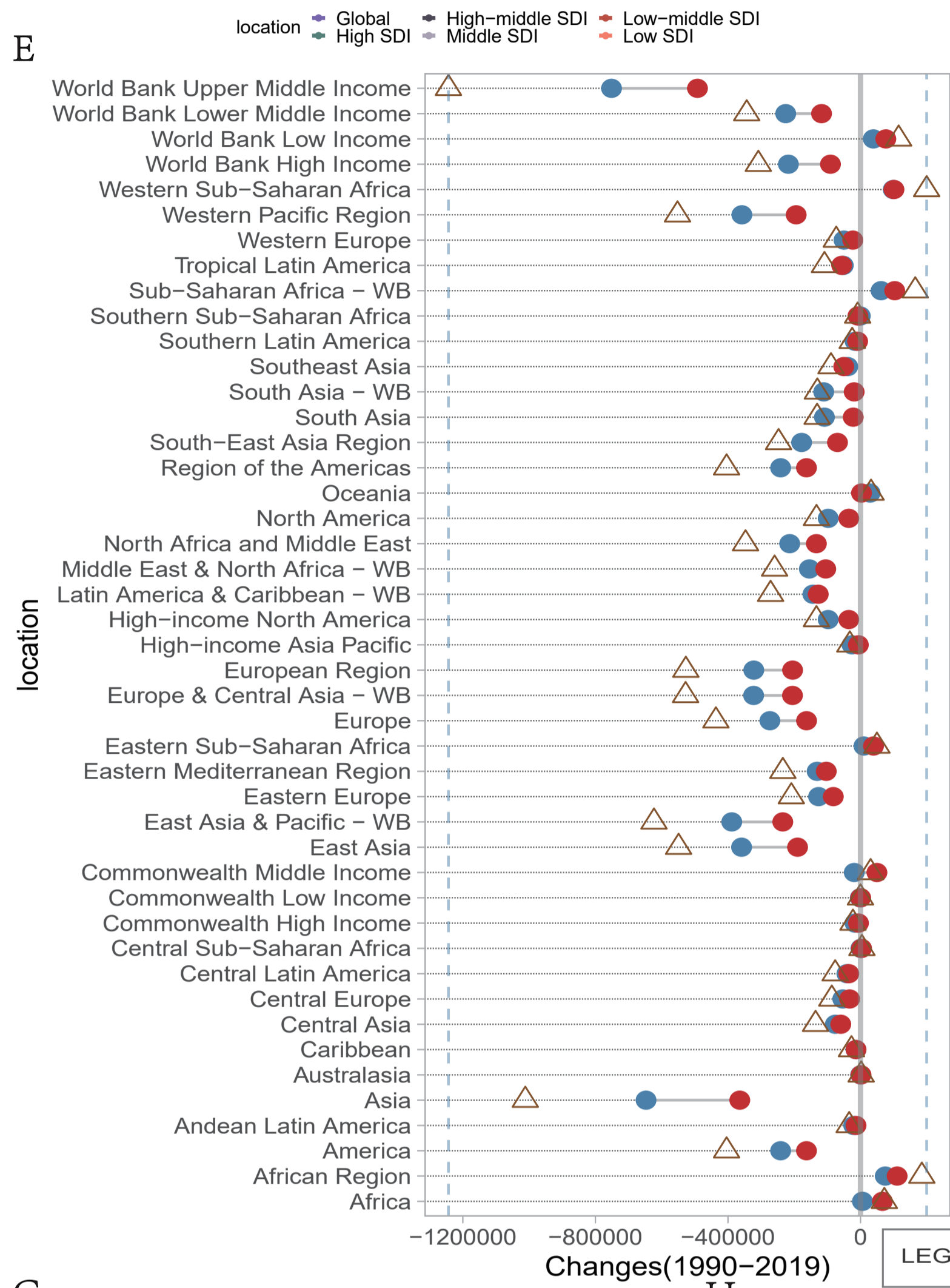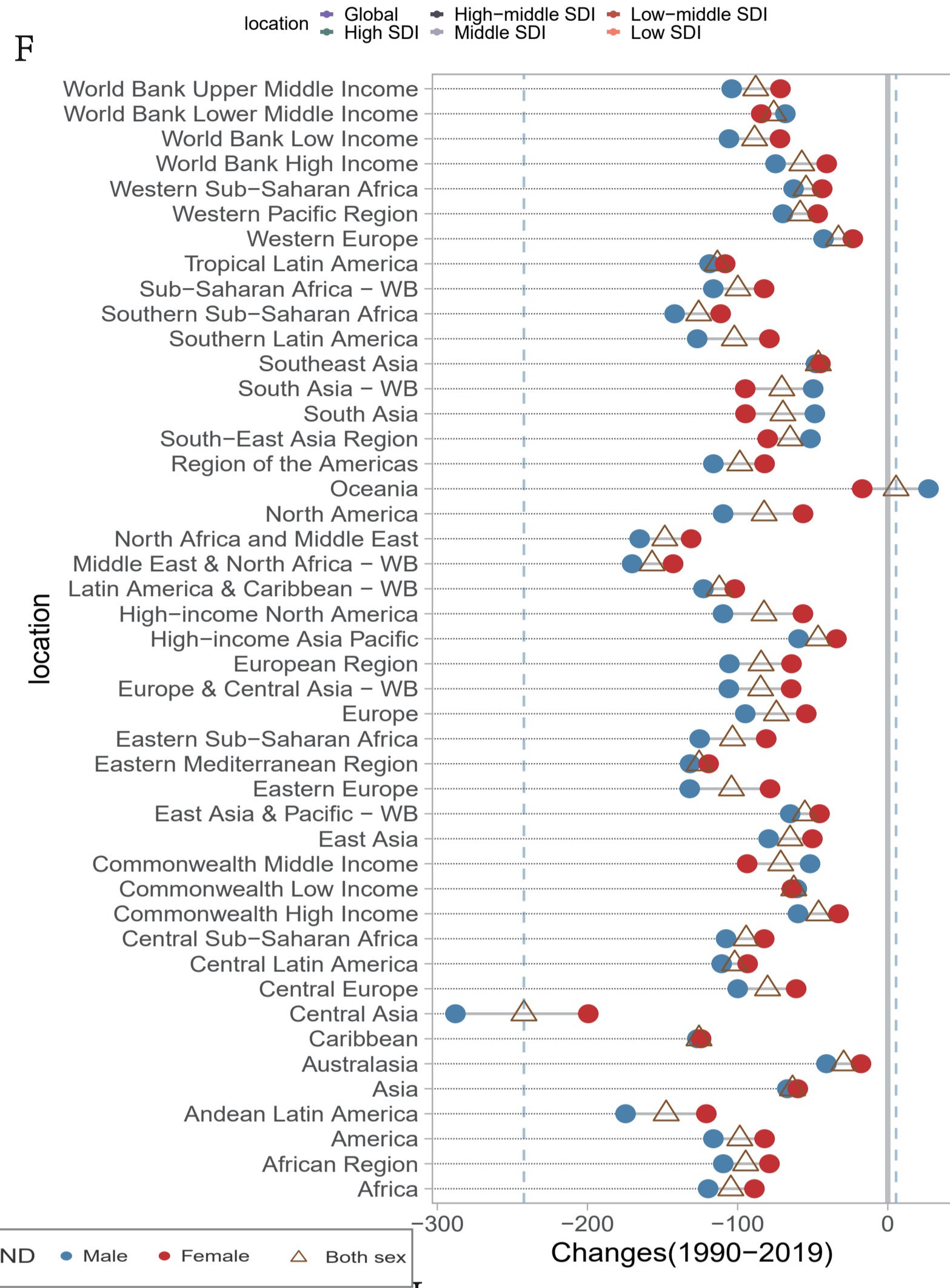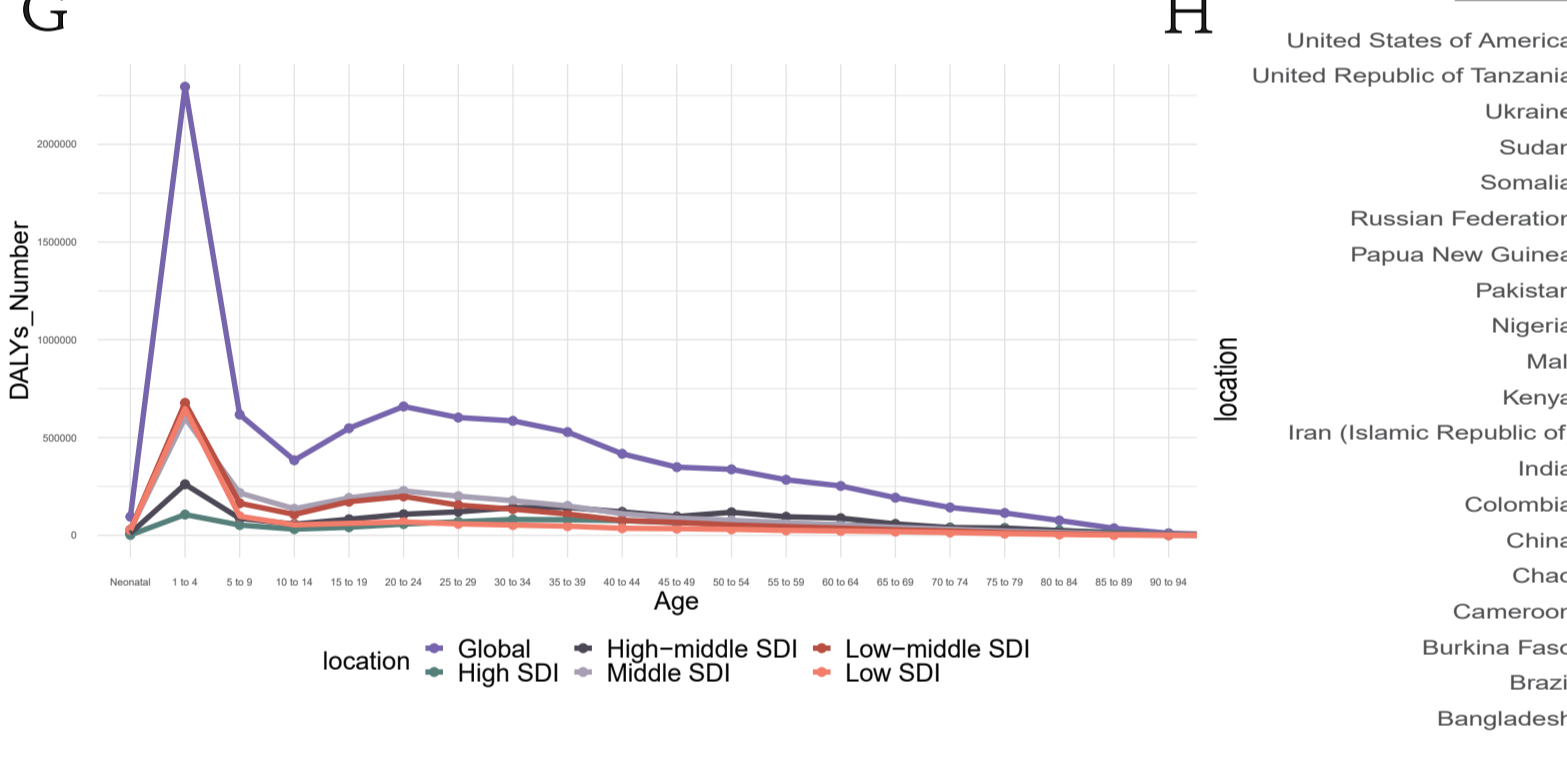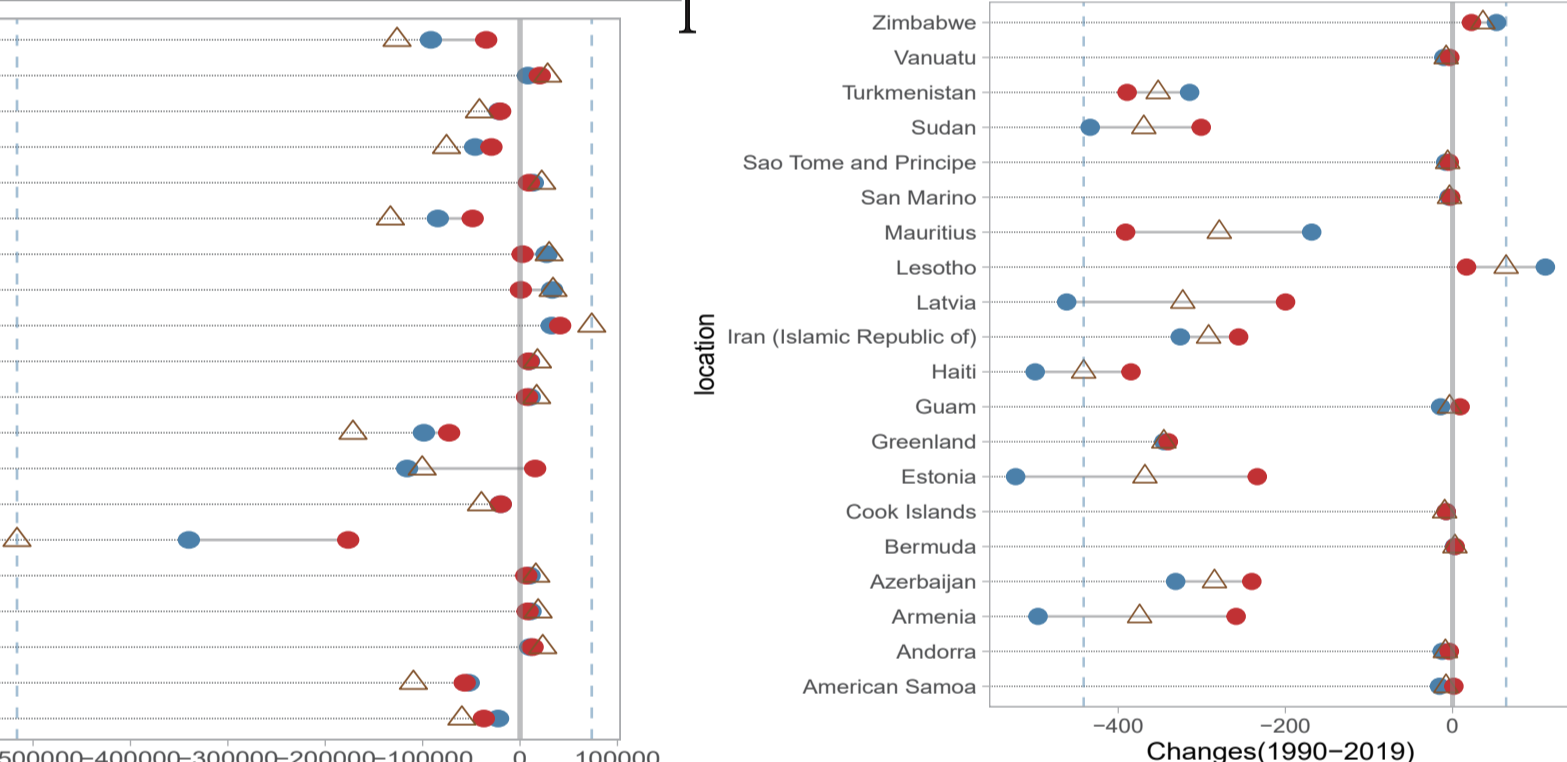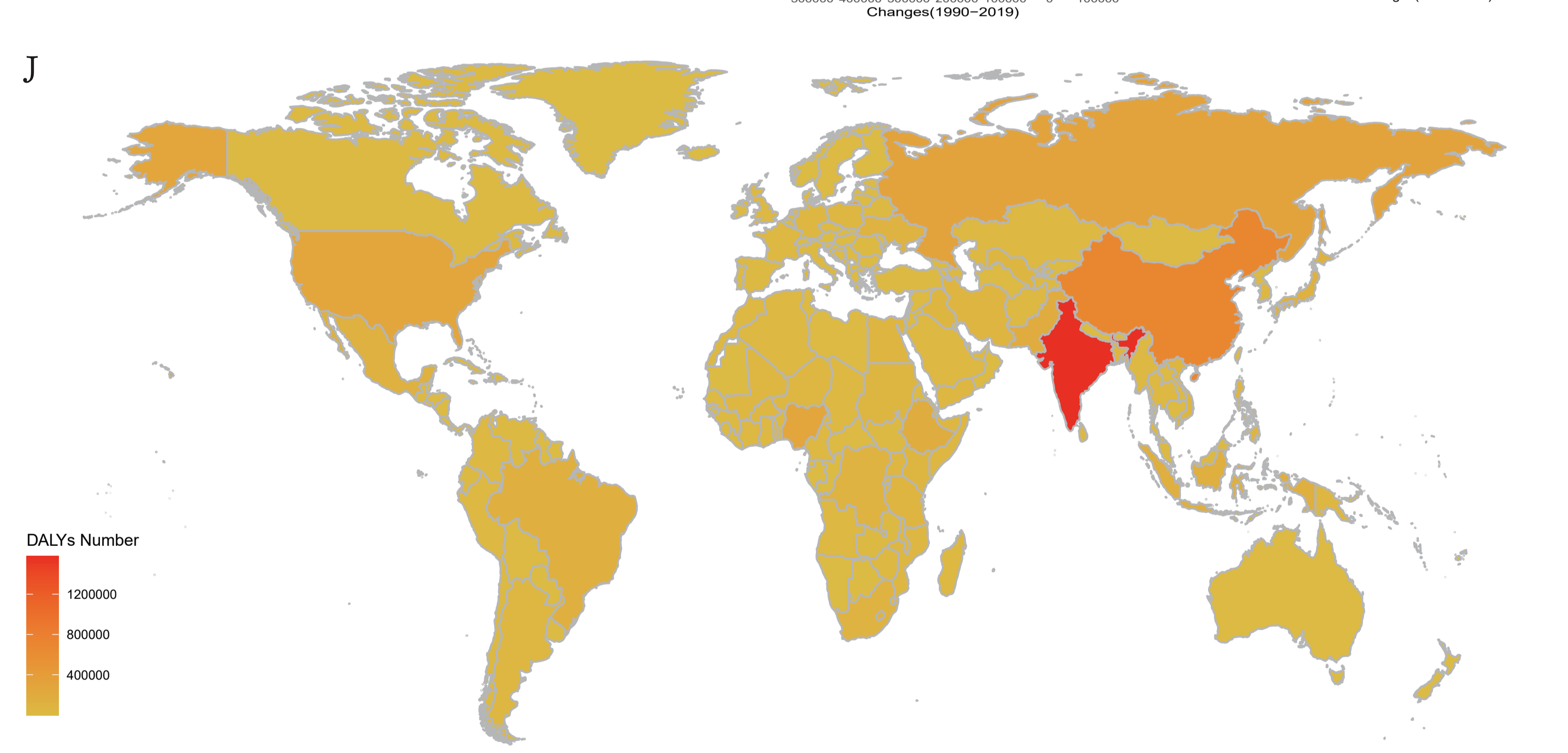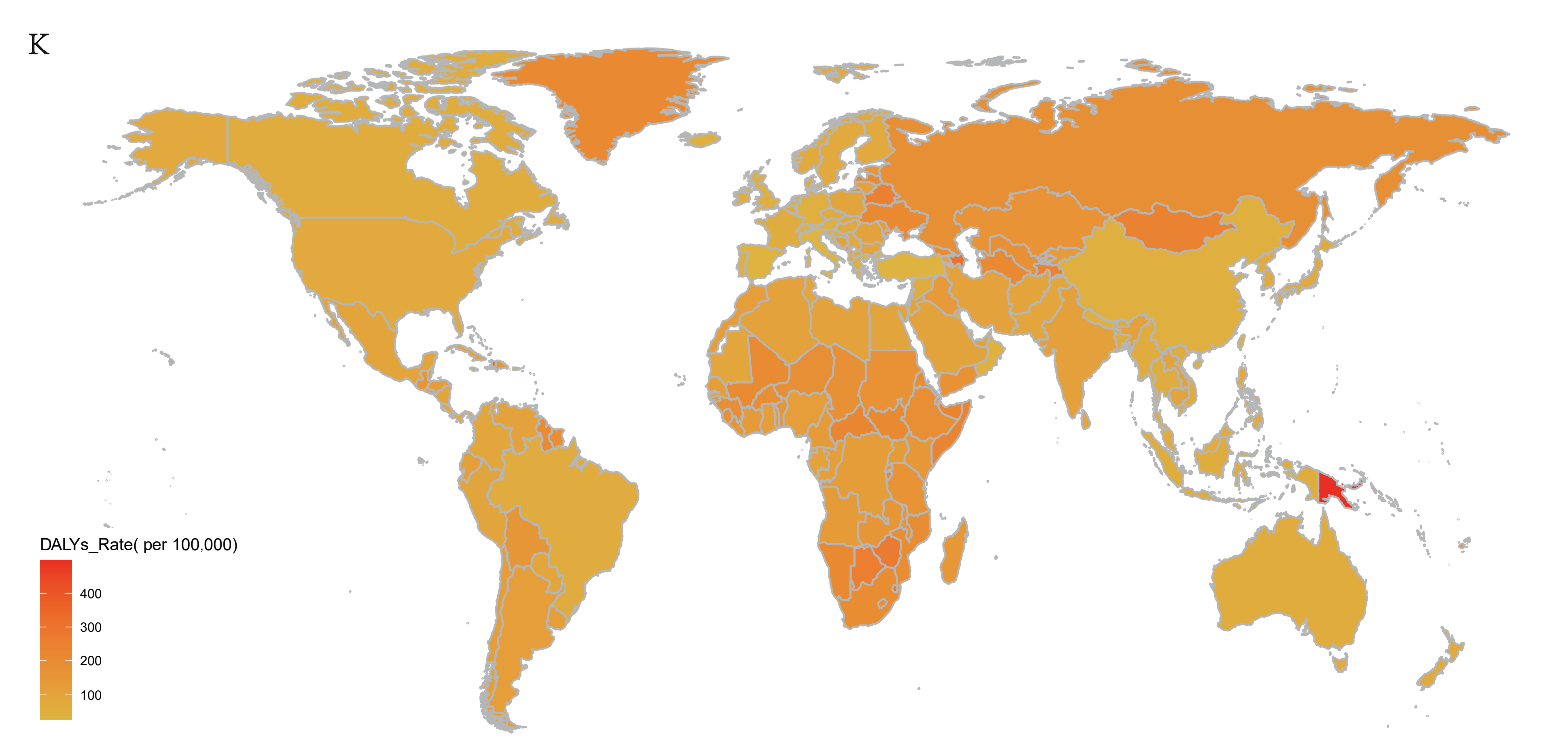

Supplement: Supplementary file 2 — Additional file 2: Supplemental Figure S2. DALYs of burns. A, the global changing trend in the number of ASDAR by sex from 1990 to 2019. B, a comparison of the ASDAR between 1990 and 2019 at global and different SDI levels. C, distribution of DALYs rate among different age categories in 2019. D, distribution of DALYs rate among different age categories in 1990. E, the range of change in DALYs by sex in 2019 compared to 1990 in 45 GBD regions. F, the range of change in ASDAR by sex in 2019 compared to 1990 in 45 GBD regions. G, the top increased or decreased in DALYs by sex in 2019 compared with 1990 among 204 countries and territories. H, the top increased or decreased in ASDAR by sex in 2019 compared with 1990 among 204 countries and territories. I, distribution of DALYs among different age categories in 1990. J, the map of DALYs in 2019 among 204 countries and territories. K, the map of ASDAR in 2019 among 204 countries and territories. DALYs, disability-adjusted life years; SDI, sociodemographic index; ASDAR, age-standardized DALYs rate; GBD, Global Burden of Disease. [file 12889_2022_13887_MOESM2_ESM.pdf]

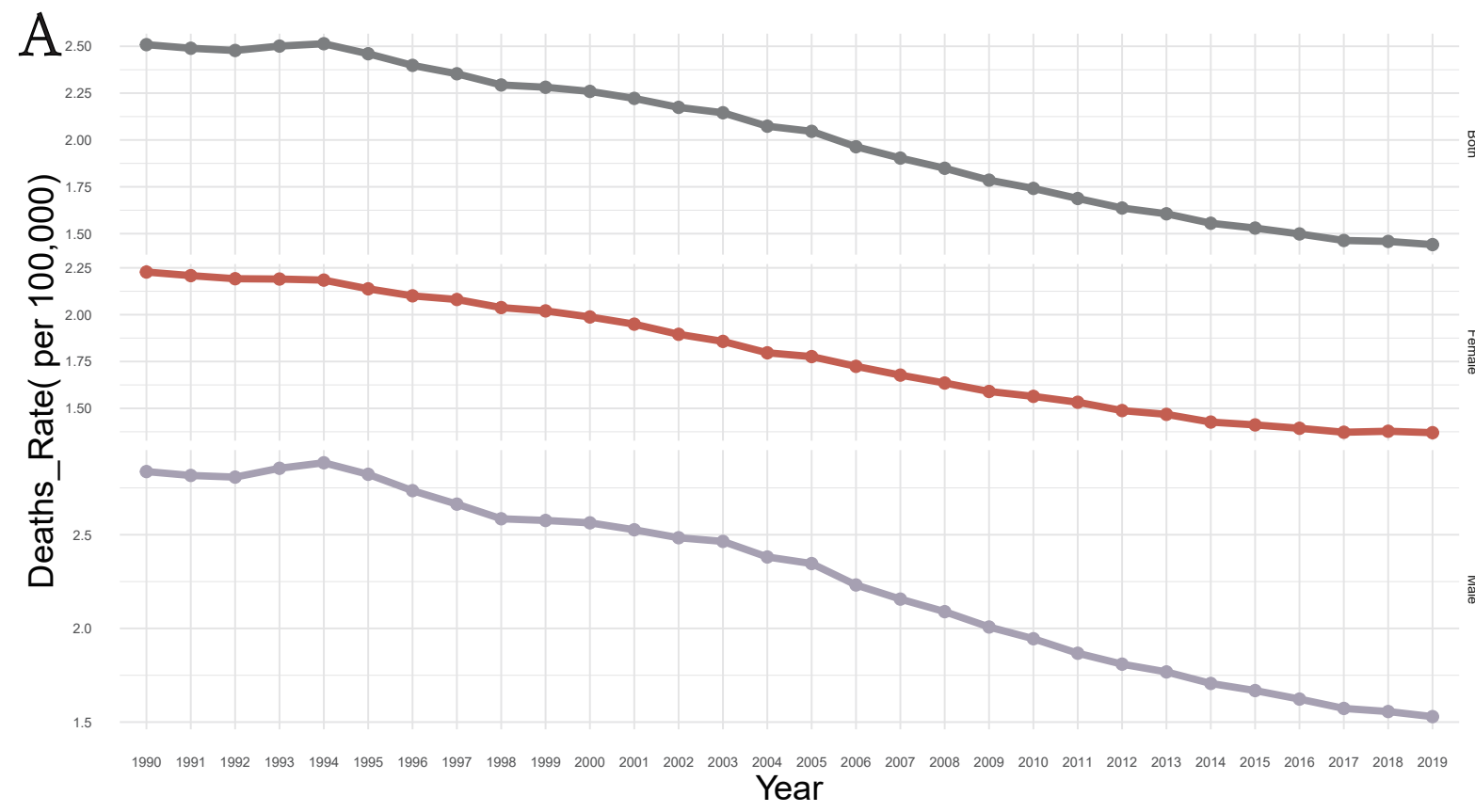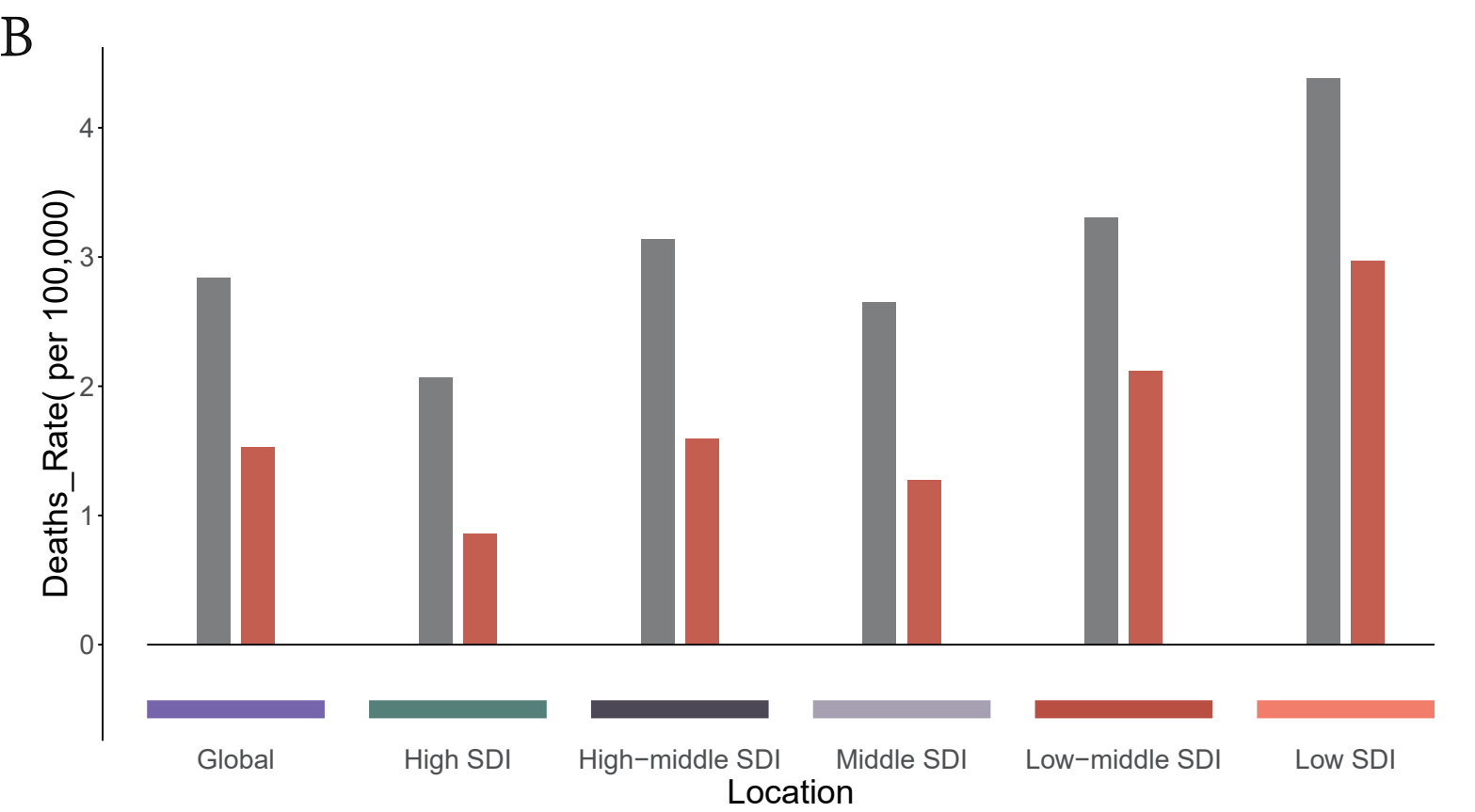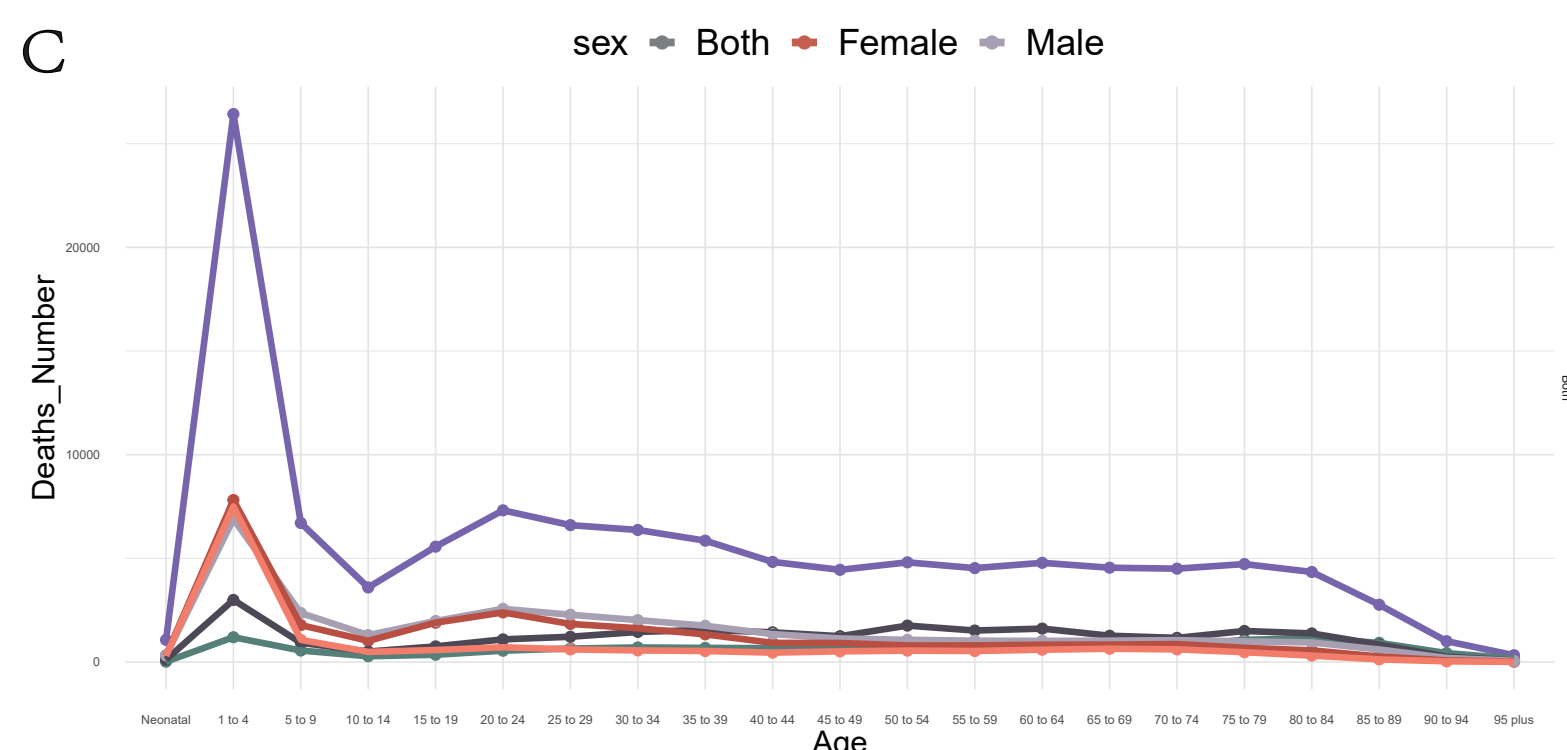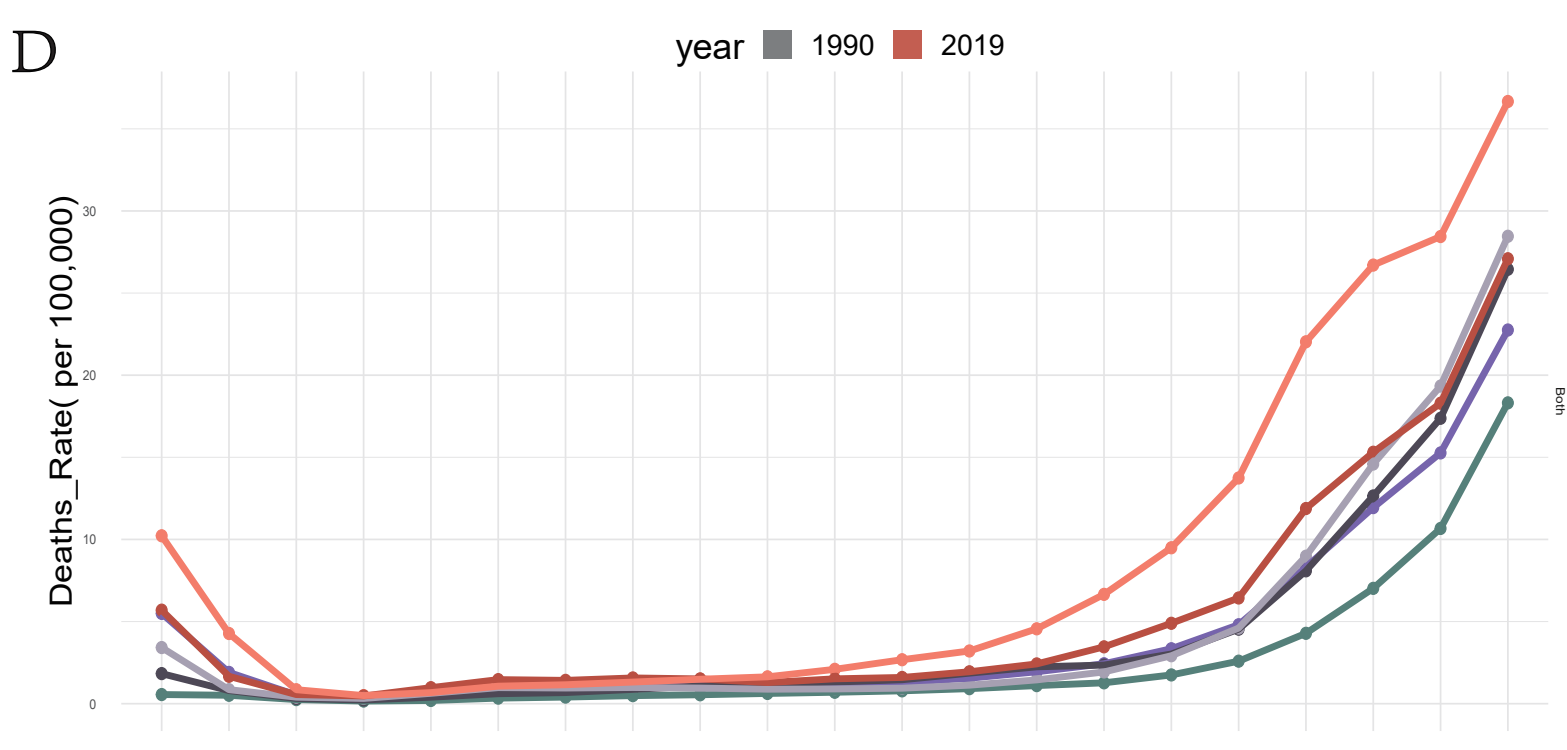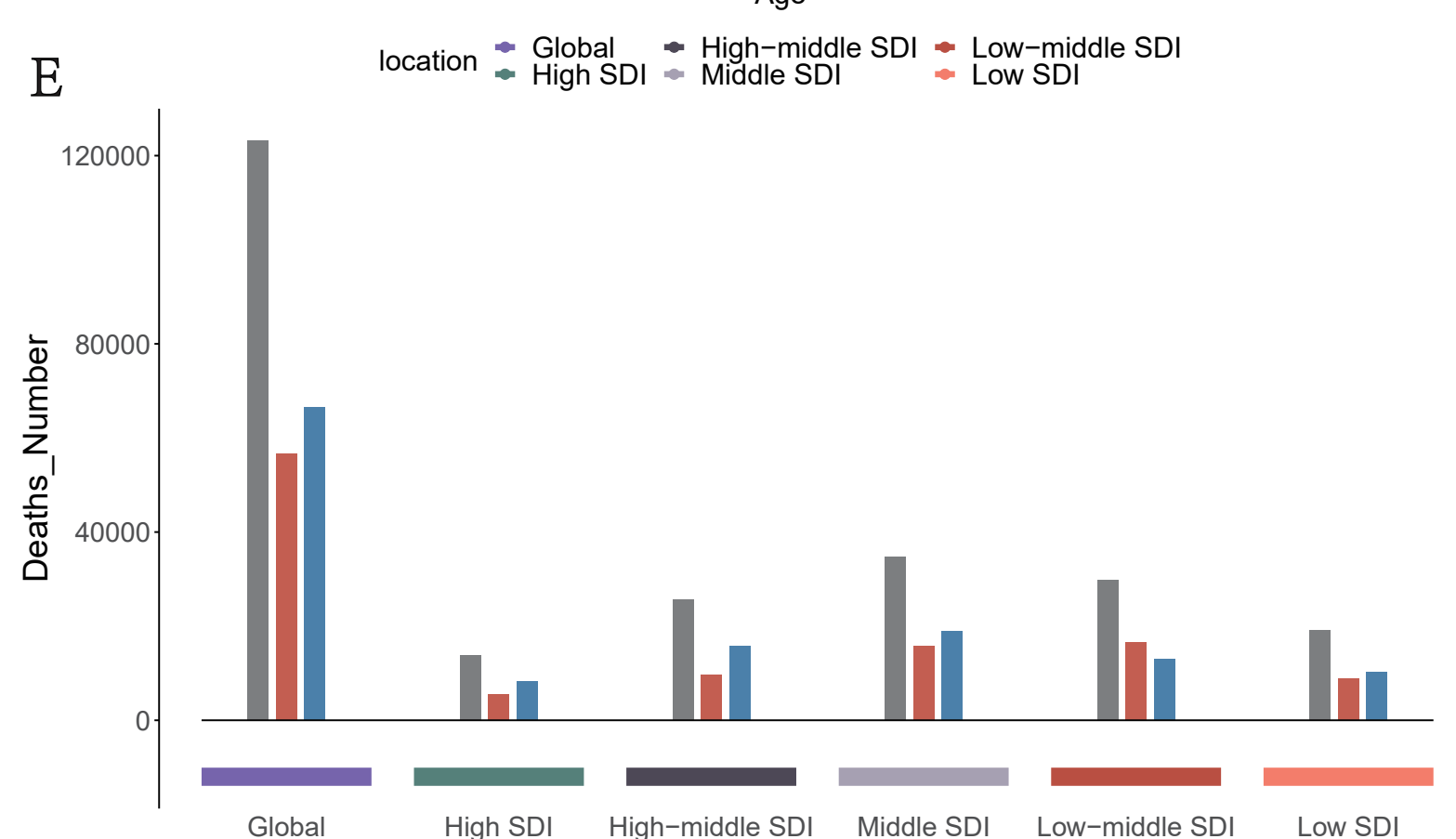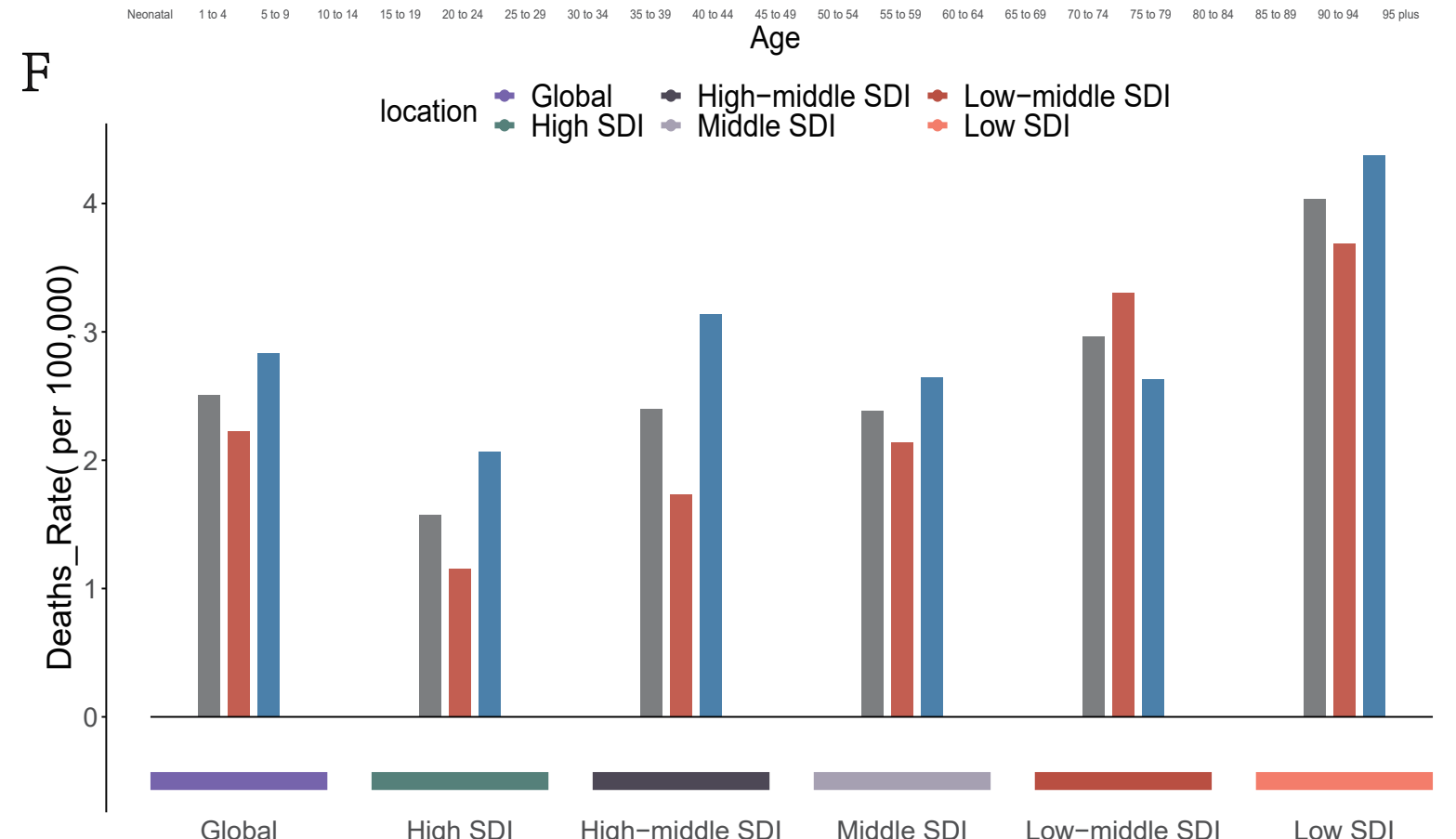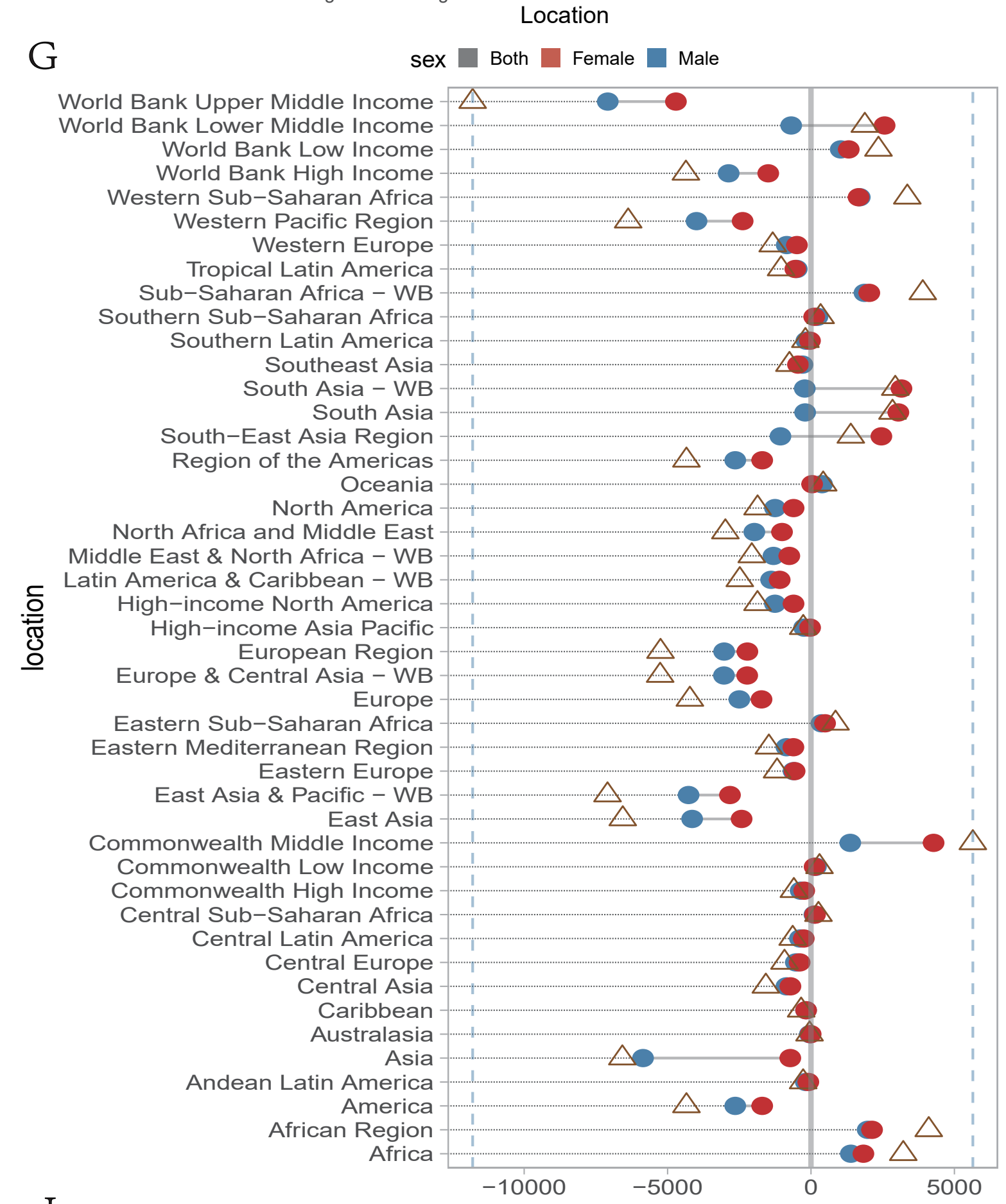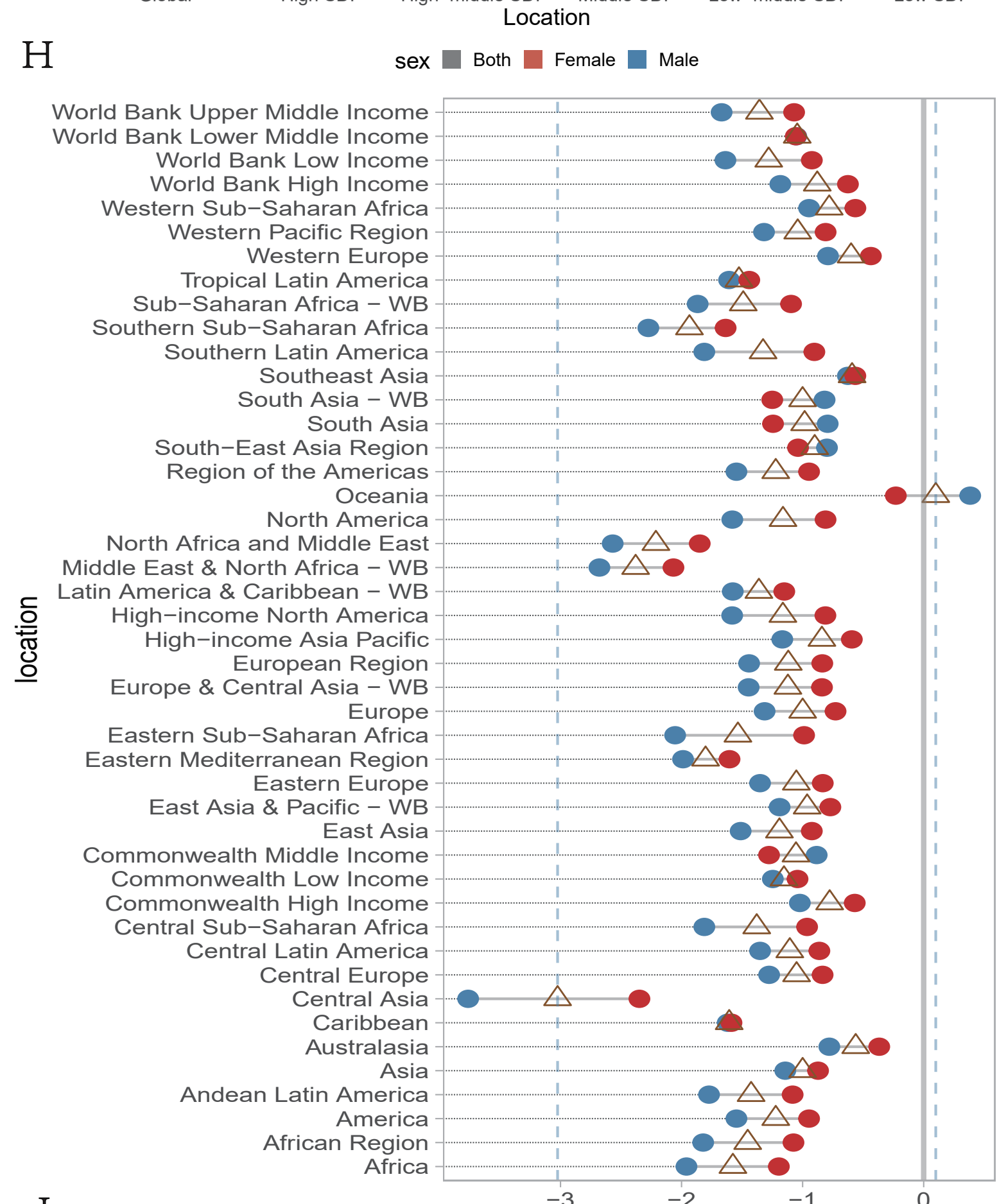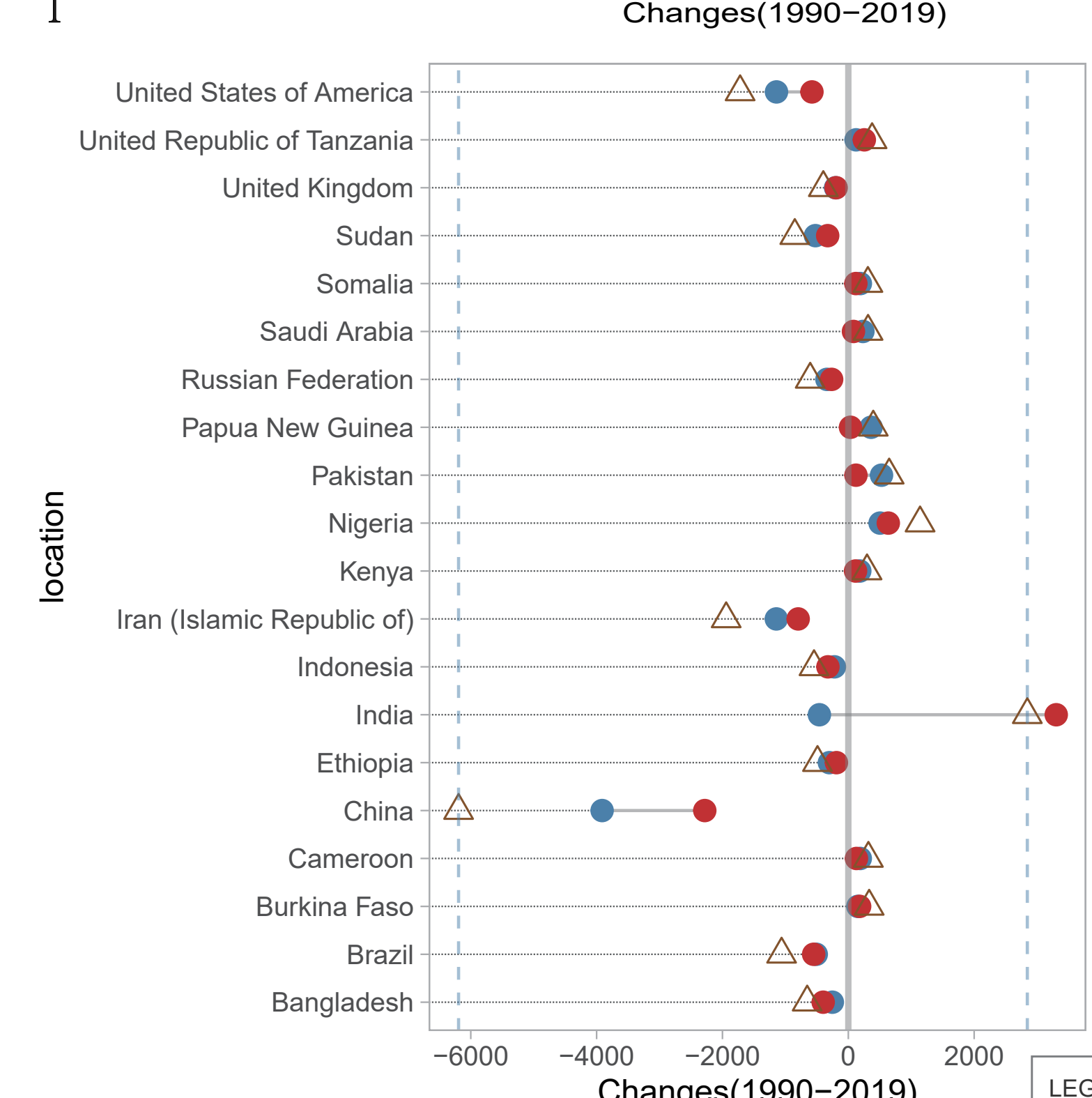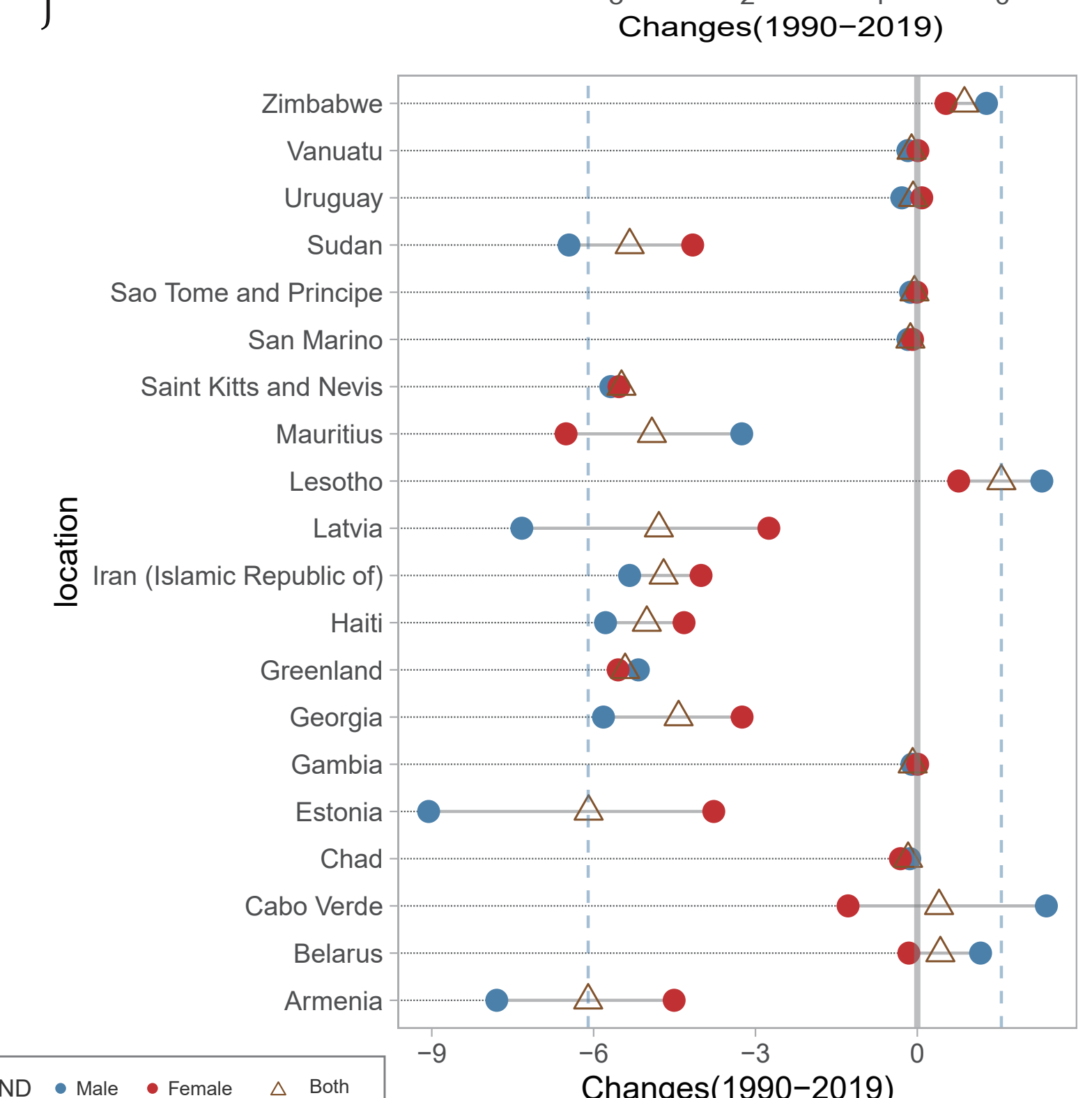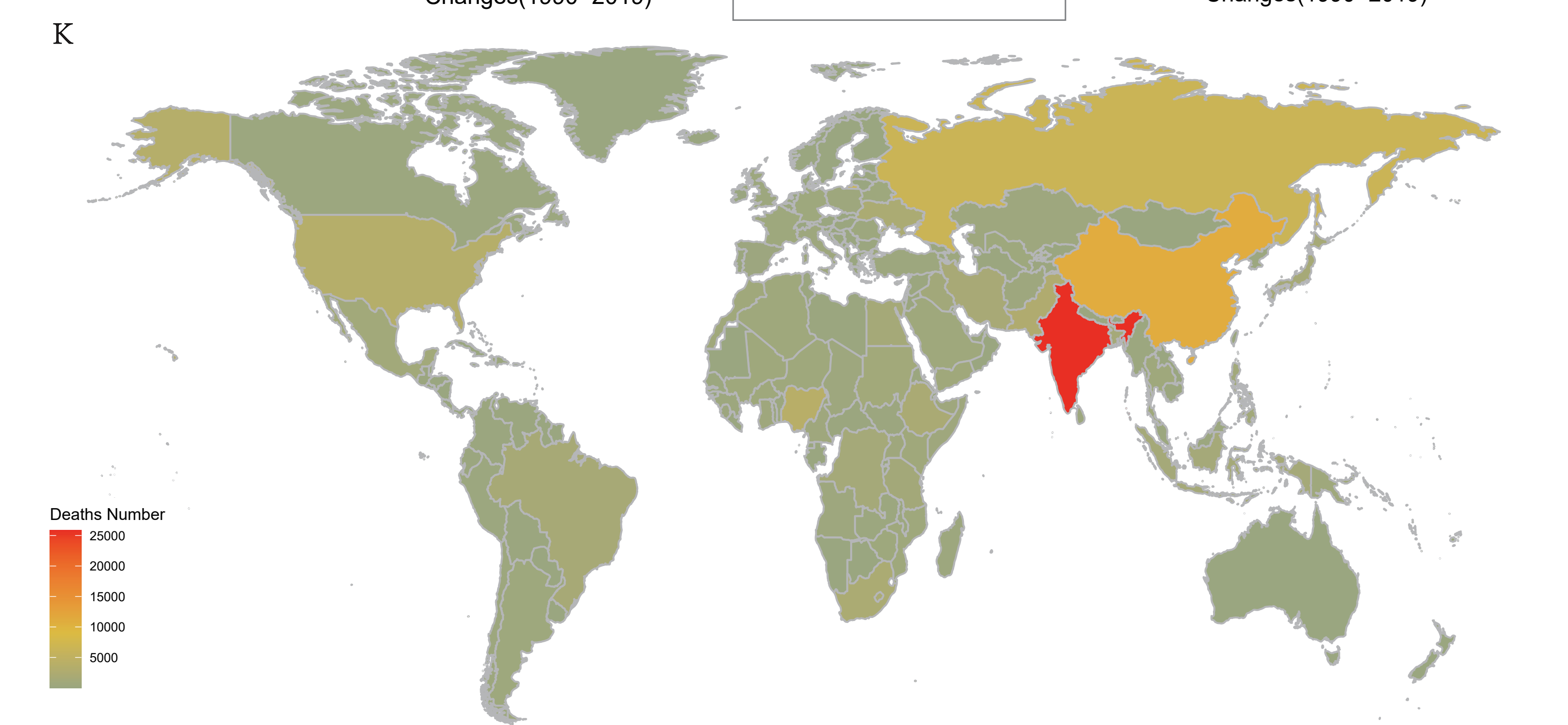

Supplement: Supplementary file 3 — Additional file 3: Supplemental Figure S3. Mortality of burns. A, the global changing trend of the ASDR by sex from 1990 to 2019. B, a comparison of ASDR between 1990 and 2019 at global and between different SDI levels. C, distribution of death rate among different age categories in 2019. D, distribution of the number of death cases among different age categories in 1990. E, a comparison of the number of death cases by sex at global and different SDI levels in 1990. F, a comparison of ASDR by sex at global and between different SDI levels in 2019. G, the range of change in the number of death cases by sex in 2019 compared with 1990 in 45 GBD regions. H, the range of change in ASDR by sex in 2019 compared with 1990 in 45 GBD regions. I, the top increased or decreased in the number of death cases by sex in 2019 compared with 1990 among 204 countries and territories. J, the top increased or decreased in ASDR by sex in 2019 compared with 1990 among 204 countries and territories. K, the map of death cases number in 2019 among 204 countries and territories. SDI, sociodemographic index; ASDR, age-standardized death rate; GBD, Global Burden of Disease. [file 12889_2022_13887_MOESM3_ESM.pdf]

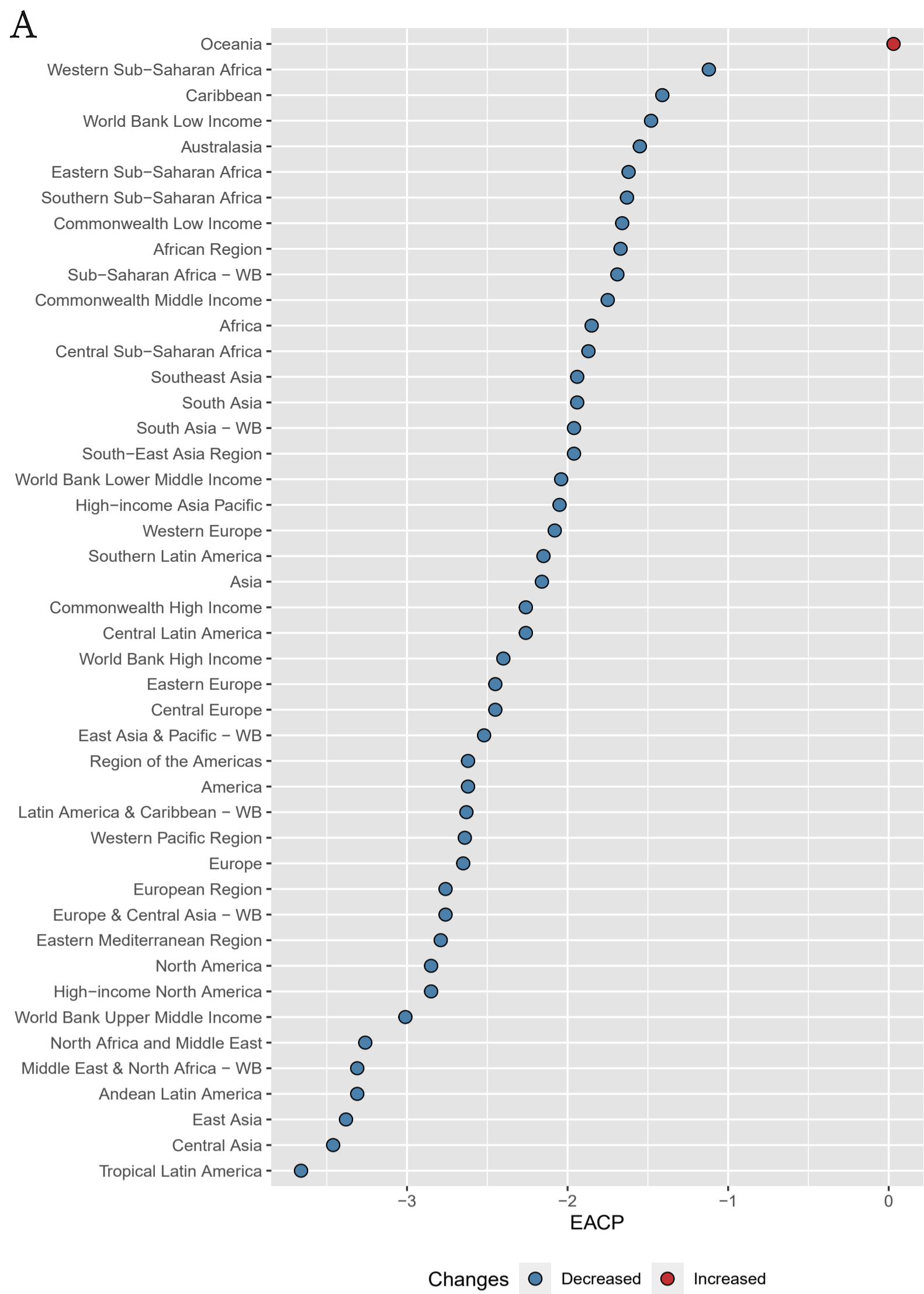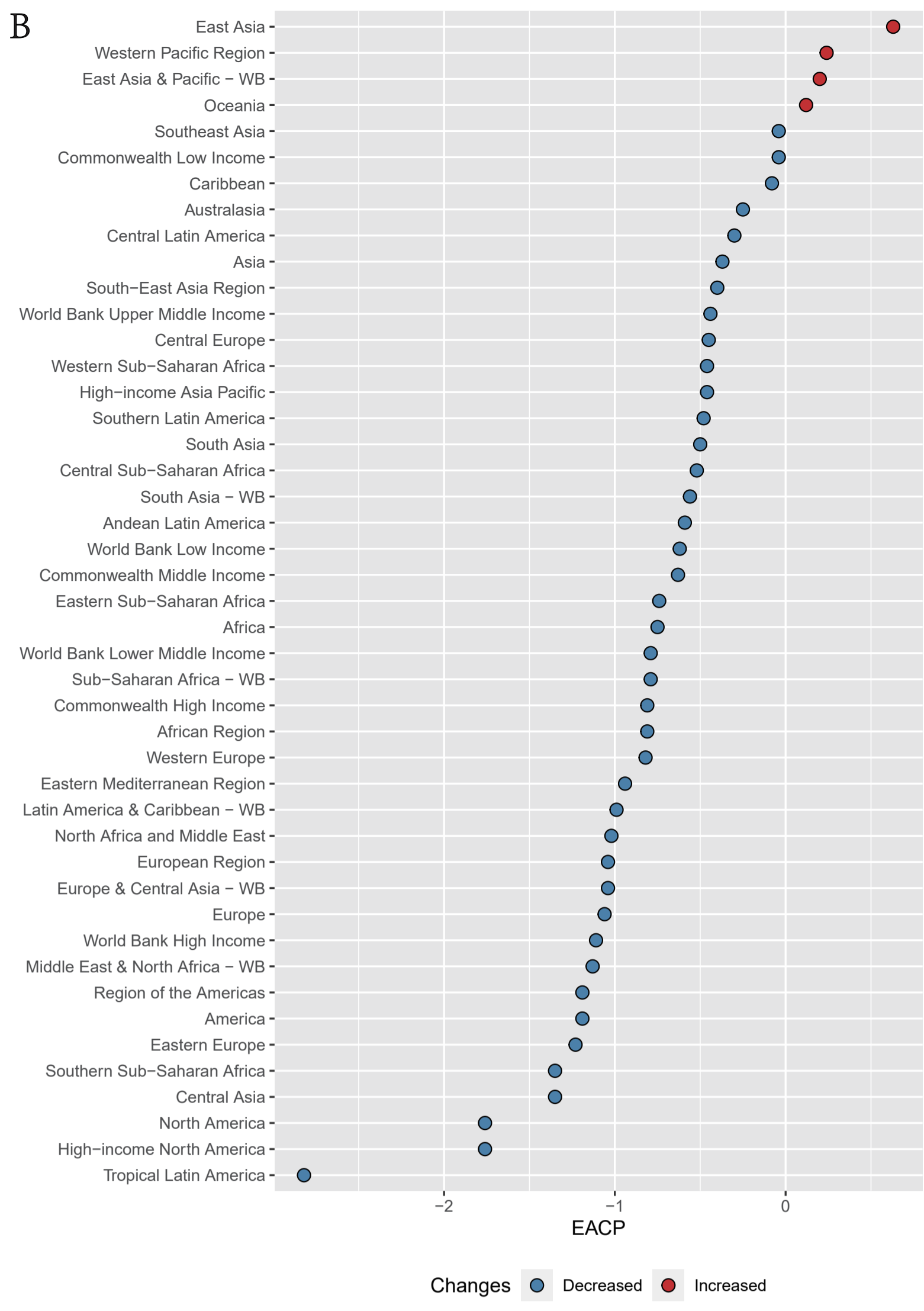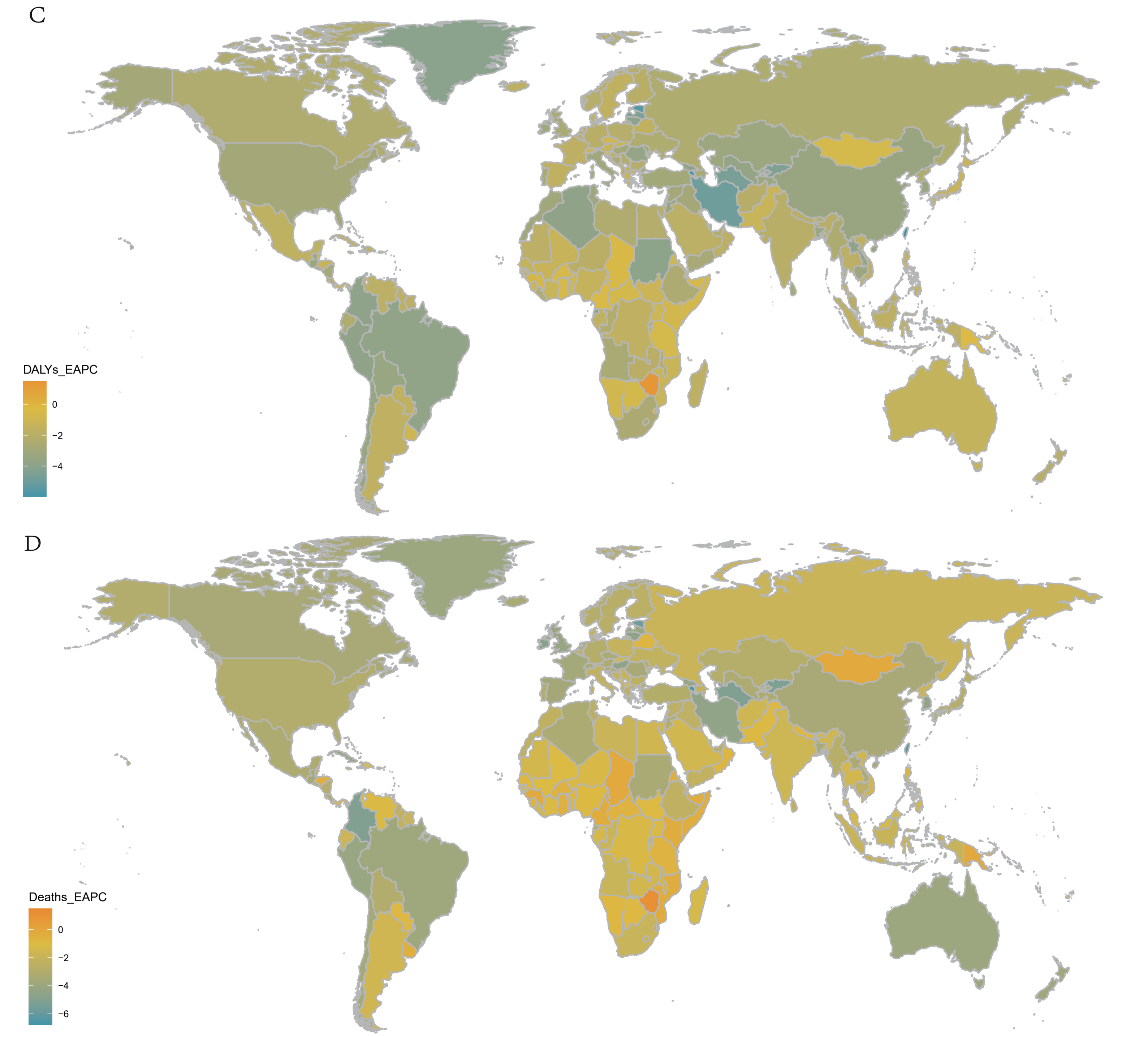

Supplement: Supplementary file 4 — Additional file 4: Supplemental Figure S4. Temporal Trends of burns. A, the EAPC of incidence in 45 GBD regions. B, the EAPC of deaths in 45 GBD regions. C, the map of deaths EAPC in 2019 among 204 countries and territories. D, the map of DALYs EAPC in 2019 among 204 countries and territories. EAPC, estimated annual percentage change; DALYs, disability-adjusted life years; GBD, Global Burden of Disease. [file 12889_2022_13887_MOESM4_ESM.pdf]

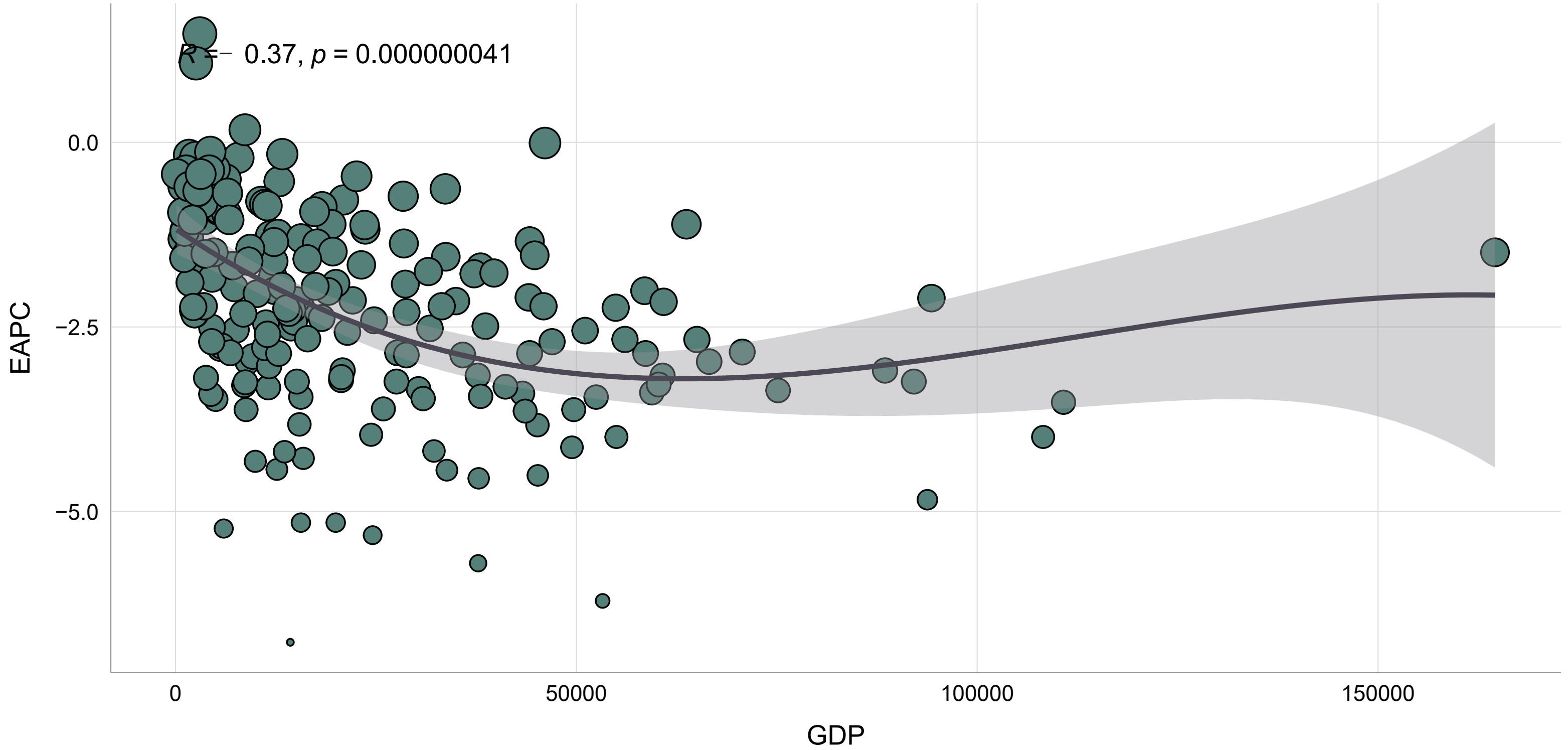

Deaths EAPC

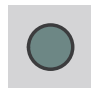

-9~-4

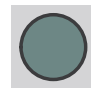

-4~1

Supplement: Supplementary file 5 — Additional file 5: Supplemental Figure S5. Relationship of EAPCs in burns incidence, DALYs, and death with SDI, UHC, and GDP. A, correlation analysis of the EAPC of ASDR with GDP. EAPC, estimated annual percentage change; DALYs, disability-adjusted life years; SDI, sociodemographic index; UHC, universal health coverage; GDP, gross domestic product; ASDR, age-standardized death rate. [file 12889_2022_13887_MOESM5_ESM.pdf]
