# Supplementary material for: The epidemiological characteristic and trends of burns globally
Source: BMC Public Health. 2022 Aug 22;22:1596. doi: 10.1186/s12889-022-13887-2 (PMC9396832; doi:10.1186/s12889-022-13887-2)
Supplement: Supplementary file 6 — Additional file 6: Table S3. GBD location hierarchy with levels (Table from:Diseases, G.B.D. and C. Injuries, Global burden of 369 diseases and injuries in 204 countries and territories, 1990-2019: a systematic analysis for the Global Burden of Disease Study 2019. Lancet (London, England), 2020. 396(10258): p. 1204-1222. ). [file 12889_2022_13887_MOESM6_ESM.pdf]

Table S3. GBD location hierarchy with levels	
**Geography**	**level**
Global	0
Low SDI	1
Low-middle SDI	1
Middle SDI	1
High-middle SDI	1
High SDI	1
Central Europe, eastern Europe, and central Asia	1
Central Asia	2
Armenia	3
Azerbaijan	3
Georgia	3
Kazakhstan	3
Kyrgyzstan	3
Mongolia	3
Tajikistan	3
Turkmenistan	3
Uzbekistan	3
Central Europe	2
Albania	3
Bosnia and Herzegovina	3
Bulgaria	3
Croatia	3
Czech Republic	3
Hungary	3
Montenegro	3
North Macedonia	3
Poland	3
Romania	3
Serbia	3
Slovakia	3
Slovenia	3
Eastern Europe	2
Belarus	3
Estonia	3
Latvia	3
Lithuania	3
Moldova	3
Russia	3
Ukraine	3
High income	1
Australasia	2
Australia	3
New Zealand	3

High-income Asia Pacific	2
Brunei	3
Japan	3
Aichi	4
Akita	4
Aomori	4
Chiba	4
Ehime	4
Fukui	4
Fukuoka	4
Fukushima	4
Gifu	4
Gunma	4
Hiroshima	4
Hokkaidō	4
Hyōgo	4
Ibaraki	4
Ishikawa	4
Iwate	4
Kagawa	4
Kagoshima	4
Kanagawa	4
Kōchi	4
Kumamoto	4
Kyōto	4
Mie	4
Miyagi	4
Miyazaki	4
Nagano	4
Nagasaki	4
Nara	4
Niigata	4
Ōita	4
Okayama	4
Okinawa	4
Ōsaka	4
Saga	4
Saitama	4
Shiga	4
Shimane	4
Shizuoka	4
Tochigi	4
Tokushima	4
Tōkyō	4
Tottori	4

Toyama	4
Wakayama	4
Yamagata	4
Yamaguchi	4
Yamanashi	4
South Korea	3
Singapore	3
High-income North America	2
Canada	3
Greenland	3
USA	3
Alabama	4
Alaska	4
Arizona	4
Arkansas	4
California	4
Colorado	4
Connecticut	4
Delaware	4
Washington, DC	4
Florida	4
Georgia	4
Hawaii	4
Idaho	4
Illinois	4
Indiana	4
Iowa	4
Kansas	4
Kentucky	4
Louisiana	4
Maine	4
Maryland	4
Massachusetts	4
Michigan	4
Minnesota	4
Mississippi	4
Missouri	4
Montana	4
Nebraska	4
Nevada	4
New Hampshire	4
New Jersey	4
New Mexico	4
New York	4
North Carolina	4

North Dakota	4
Ohio	4
Oklahoma	4
Oregon	4
Pennsylvania	4
Rhode Island	4
South Carolina	4
South Dakota	4
Tennessee	4
Texas	4
Utah	4
Vermont	4
Virginia	4
Washington	4
West Virginia	4
Wisconsin	4
Wyoming	4
Southern Latin America	2
Argentina	3
Chile	3
Uruguay	3
Western Europe	2
Andorra	3
Austria	3
Belgium	3
Cyprus	3
Denmark	3
Finland	3
France	3
Germany	3
Greece	3
Iceland	3
Ireland	3
Israel	3
Italy	3
Luxembourg	3
Malta	3
Monaco	3
Netherlands	3
Norway	3
Portugal	3
San Marino	3
Spain	3
Sweden	3
Stockholm	4

Sweden except Stockholm	4
Switzerland	3
UK	3
England	4
East Midlands	5
Derby	6
Derbyshire	6
Leicester	6
Leicestershire	6
Lincolnshire	6
Northamptonshire	6
Nottingham	6
Nottinghamshire	6
Rutland	6
East of England	5
Bedford	6
Cambridgeshire	6
Central Bedfordshire	6
Essex	6
Hertfordshire	6
Luton	6
Norfolk	6
Peterborough	6
Southend-on-Sea	6
Suffolk	6
Thurrock	6
Greater London	5
Barking and Dagenham	6
Barnet	6
Bexley	6
Brent	6
Bromley	6
Camden	6
Croydon	6
Ealing	6
Enfield	6
Greenwich	6
Hackney	6
Hammersmith and Fulham	6
Haringey	6
Harrow	6
Havering	6
Hillingdon	6
Hounslow	6
Islington	6

Kensington and Chelsea	6
Kingston upon Thames	6
Lambeth	6
Lewisham	6
Merton	6
Newham	6
Redbridge	6
Richmond upon Thames	6
Southwark	6
Sutton	6
Tower Hamlets	6
Waltham Forest	6
Wandsworth	6
Westminster	6
North East England	5
County Durham	6
Darlington	6
Gateshead	6
Hartlepool	6
Middlesbrough	6
Newcastle upon Tyne	6
North Tyneside	6
Northumberland	6
Redcar and Cleveland	6
South Tyneside	6
Stockton-on-Tees	6
Sunderland	6
North West England	5
Blackburn with Darwen	6
Blackpool	6
Bolton	6
Bury	6
Cheshire East	6
Cheshire West and Chester	6
Cumbria	6
Halton	6
Knowsley	6
Lancashire	6
Liverpool	6
Manchester	6
Oldham	6
Rochdale	6
Salford	6
Sefton	6
St Helens	6

Stockport	6
Tameside	6
Trafford	6
Warrington	6
Wigan	6
Wirral	6
South East England	5
Bracknell Forest	6
Brighton and Hove	6
Buckinghamshire	6
East Sussex	6
Hampshire	6
Isle of Wight	6
Kent	6
Medway	6
Milton Keynes	6
Oxfordshire	6
Portsmouth	6
Reading	6
Slough	6
Southampton	6
Surrey	6
West Berkshire	6
West Sussex	6
Windsor and Maidenhead	6
Wokingham	6
South West England	5
Bath and North East Somerset	6
Bournemouth	6
Bristol, City of	6
Cornwall	6
Devon	6
Dorset	6
Gloucestershire	6
North Somerset	6
Plymouth	6
Poole	6
Somerset	6
South Gloucestershire	6
Swindon	6
Torbay	6
Wiltshire	6
West Midlands	5
Birmingham	6
Coventry	6

Dudley	6
Herefordshire, County of	6
Sandwell	6
Shropshire	6
Solihull	6
Staffordshire	6
Stoke-on-Trent	6
Telford and Wrekin	6
Walsall	6
Warwickshire	6
Wolverhampton	6
Worcestershire	6
Yorkshire and the Humber	5
Barnsley	6
Bradford	6
Calderdale	6
Doncaster	6
East Riding of Yorkshire	6
Kingston upon Hull, City of	6
Kirklees	6
Leeds	6
North East Lincolnshire	6
North Lincolnshire	6
North Yorkshire	6
Rotherham	6
Sheffield	6
Wakefield	6
York	6
Northern Ireland	4
Scotland	4
Wales	4
Latin America and Caribbean	1
Andean Latin America	2
Bolivia	3
Ecuador	3
Peru	3
Caribbean	2
Antigua and Barbuda	3
The Bahamas	3
Barbados	3
Belize	3
Bermuda	3
Cuba	3
Dominica	3
Dominican Republic	3

Grenada	3
Guyana	3
Haiti	3
Jamaica	3
Puerto Rico	3
Saint Kitts and Nevis	3
Saint Lucia	3
Saint Vincent and the Grenadines	3
Suriname	3
Trinidad and Tobago	3
Virgin Islands	3
Central Latin America	2
Colombia	3
Costa Rica	3
El Salvador	3
Guatemala	3
Honduras	3
Mexico	3
Aguascalientes	4
Baja California	4
Baja California Sur	4
Campeche	4
Chiapas	4
Chihuahua	4
Coahuila	4
Colima	4
Durango	4
Guanajuato	4
Guerrero	4
Hidalgo	4
Jalisco	4
México	4
Mexico City	4
Michoacán de Ocampo	4
Morelos	4
Nayarit	4
Nuevo León	4
Oaxaca	4
Puebla	4
Querétaro	4
Quintana Roo	4
San Luis Potosí	4
Sinaloa	4
Sonora	4
Tabasco	4

Tamaulipas	4
Tlaxcala	4
Veracruz de Ignacio de la Llave	4
Yucatán	4
Zacatecas	4
Nicaragua	3
Panama	3
Venezuela	3
Tropical Latin America	2
Brazil	3
Acre	4
Alagoas	4
Amapá	4
Amazonas	4
Bahia	4
Ceará	4
Distrito Federal	4
Espírito Santo	4
Goiás	4
Maranhão	4
Mato Grosso	4
Mato Grosso do Sul	4
Minas Gerais	4
Pará	4
Paraíba	4
Paraná	4
Pernambuco	4
Piauí	4
Rio de Janeiro	4
Rio Grande do Norte	4
Rio Grande do Sul	4
Rondônia	4
Roraima	4
Santa Catarina	4
São Paulo	4
Sergipe	4
Tocantins	4
Paraguay	3
North Africa and Middle East	1
North Africa and Middle East	2
Afghanistan	3
Algeria	3
Bahrain	3
Egypt	3
Iran	3

Iraq	3
Jordan	3
Kuwait	3
Lebanon	3
Libya	3
Morocco	3
Oman	3
Palestine	3
Qatar	3
Saudi Arabia	3
Sudan	3
Syria	3
Tunisia	3
Turkey	3
United Arab Emirates	3
Yemen	3
South Asia	1
South Asia	2
Bangladesh	3
Bhutan	3
India	3
Andhra Pradesh	4
Arunachal Pradesh	4
Assam	4
Bihar C	4
hhattisgarh	4
Delhi	4
Goa	4
Gujarat	4
Haryana	4
Himachal Pradesh	4
Jammu & Kashmir and Ladakh	4
Jharkhand	4
Karnataka	4
Kerala	4
Madhya Pradesh	4
Maharashtra	4
Manipur	4
Meghalaya	4
Mizoram	4
Nagaland	4
Odisha	4
Punjab	4
Rajasthan	4
Sikkim	4

Tamil Nadu	4
Telangana	4
Tripura	4
Other Union Territories	4
Uttar Pradesh	4
Uttarakhand	4
West Bengal	4
Nepal	3
Pakistan	3
Southeast Asia, east Asia, and Oceania	1
East Asia	2
China	3
North Korea	3
Taiwan (province of China)	3
Oceania	2
American Samoa	3
Cook Islands	3
Fiji	3
Guam	3
Kiribati	3
Marshall Islands	3
Federated States of Micronesia	3
Nauru	3
Niue	3
Northern Mariana Islands	3
Palau	3
Papua New Guinea	3
Samoa	3
Solomon Islands	3
Tokelau	3
Tonga	3
Tuvalu	3
Vanuatu	3
Southeast Asia	2
Cambodia	3
Indonesia	3
Aceh	4
Bali	4
Bangka-Belitung Islands	4
Banten	4
Bengkulu	4
Gorontalo	4
Jakarta	4
Jambi	4
West Java	4

Central Java	4
East Java	4
West Kalimantan	4
South Kalimantan	4
Central Kalimantan	4
East Kalimantan	4
North Kalimantan	4
Riau Islands	4
Lampung	4
Maluku	4
North Maluku	4
West Nusa Tenggara	4
East Nusa Tenggara	4
Papua	4
West Papua	4
Riau	4
West Sulawesi	4
South Sulawesi	4
Central Sulawesi	4
Southeast Sulawesi	4
North Sulawesi	4
West Sumatra	4
South Sumatra	4
North Sumatra	4
Yogyakarta	4
Laos	3
Malaysia	3
Maldives	3
Mauritius	3
Myanmar	3
Philippines	3
Seychelles	3
Sri Lanka	3
Thailand	3
Timor-Leste	3
Vietnam	3
Sub-Saharan Africa	1
Central sub-Saharan Africa	2
Angola	3
Central African Republic	3
Congo (Brazzaville)	3
DR Congo	3
Equatorial Guinea	3
Gabon	3
Eastern sub-Saharan Africa	2

Burundi	3
Comoros	3
Djibouti	3
Eritrea	3
Ethiopia	3
Kenya	3
Baringo	4
Bomet	4
Bungoma	4
Busia	4
Elgeyo Marakwet	4
Embu	4
Garissa	4
Homa Bay	4
Isiolo	4
Kajiado	4
Kakamega	4
Kericho	4
Kiambu	4
Kilifi	4
Kirinyaga	4
Kisii	4
Kisumu	4
Kitui	4
Kwale	4
Laikipia	4
Lamu	4
Machakos	4
Makueni	4
Mandera	4
Marsabit	4
Meru	4
Migori	4
Mombasa	4
Murang'a	4
Nairobi	4
Nakuru	4
Nandi	4
Narok	4
Nyamira	4
Nyandarua	4
Nyeri	4
Samburu	4
Siaya	4
Taita Taveta	4

Tana River	4
Tharaka Nithi	4
Trans Nzoia	4
Turkana	4
Uasin Gishu	4
Vihiga	4
Wajir	4
West Pokot	4
Madagascar	3
Malawi	3
Mozambique	3
Rwanda	3
Somalia	3
South Sudan	3
Uganda	3
Tanzania	3
Zambia	3
Southern sub-Saharan Africa	2
Botswana	3
eSwatini	3
Lesotho	3
Namibia	3
South Africa	3
Zimbabwe	3
Western sub-Saharan Africa	2
Benin	3
Burkina Faso	3
Cape Verde	3
Cameroon	3
Chad	3
Côte d'Ivoire	3
The Gambia	3
Ghana	3
Guinea	3
Guinea-Bissau	3
Liberia	3
Mali	3
Mauritania	3
Niger	3
Nigeria	3
São Tomé and PrÍncipe	3
Senegal	3
Sierra Leone	3
Togo	3
